# ABIN1 (Q478) is Required to Prevent Hematopoietic Deficiencies through Regulating Type I IFNs Expression

**DOI:** 10.1002/advs.202303555

**Published:** 2023-11-27

**Authors:** Xuanhui Wu, Yong Wang, Bingyi Chen, Yongbo Liu, Fang Li, Yangjing Ou, Haiwei Zhang, Xiaoxia Wu, Xiaoming Li, Lingxia Wang, Wuwei Rong, Jianling Liu, Mingyan Xing, Xiaoming Zhao, Han Liu, Lingling Ge, Ankang Lv, Lan Wang, Zhichao Wang, Ming Li, Haibing Zhang

**Affiliations:** ^1^ CAS Key Laboratory of Nutrition, Metabolism and Food Safety Shanghai Institute of Nutrition and Health University of Chinese Academy of Sciences Chinese Academy of Sciences Shanghai 200031 China; ^2^ Department of Anesthesiology Shanghai First People's Hospital Shanghai Jiaotong University Shanghai 200080 China; ^3^ Department of Cardiology Ruijin Hospital Shanghai Jiaotong University School of Medicine Shanghai 200025 China; ^4^ Department of Plastic and Reconstructive Surgery Shanghai Ninth People's Hospital Shanghai Jiao Tong University School of Medicine Shanghai 200011 China

**Keywords:** ABIN1, ABIN1(Q478), IFN‐I, RIPK3, MDS‐liked diseases

## Abstract

A20‐binding inhibitor of NF‐κB activation (ABIN1) is a polyubiquitin‐binding protein that regulates cell death and immune responses. Although *Abin1* is located on chromosome 5q in the region commonly deleted in patients with 5q minus syndrome, the most distinct of the myelodysplastic syndromes (MDSs), the precise role of ABIN1 in MDSs remains unknown. In this study, mice with a mutation disrupting the polyubiquitin‐binding site (*Abin1^Q478H/Q478H^*) is generated. These mice develop MDS‐like diseases characterized by anemia, thrombocytopenia, and megakaryocyte dysplasia. Extramedullary hematopoiesis and bone marrow failure are also observed in *Abin1^Q478H/Q478H^* mice. Although *Abin1^Q478H/Q478H^* cells are sensitive to RIPK1 kinase–RIPK3–MLKL‐dependent necroptosis, only anemia and splenomegaly are alleviated by RIPK3 deficiency but not by MLKL deficiency or the RIPK1 kinase‐dead mutation. This indicates that the necroptosis‐independent function of RIPK3 is critical for anemia development in *Abin1^Q478H/Q478H^* mice. Notably, Abin1^Q478H/Q478H^ mice exhibit higher levels of type I interferon (IFN‐I) expression in bone marrow cells compared towild‐type mice. Consistently, blocking type I IFN signaling through the co‐deletion of *Ifnar1* greatly ameliorated anemia, thrombocytopenia, and splenomegaly in *Abin1^Q478H/Q478H^* mice. Together, these results demonstrates that ABIN1(Q478) prevents the development of hematopoietic deficiencies by regulating type I IFN expression.

## Graphical abstract

The mice expressing the Q478H mutation in the polyubiquitin‐binding domain of ABIN1 (*Abin1^Q478H/Q478H^* mice) exhibit MDS‐like diseases, including anemia, thrombocytopenia, and megakaryocyte dysplasia. Necroptosis‐independent RIPK3 function contributes to anemia, while co‐deletion of *Ifnar1* rescues anemia, thrombocytopenia, and splenomegaly, highlighting ABIN1 (Q478) as a regulator of hematopoietic deficiencies through type I IFN expression.

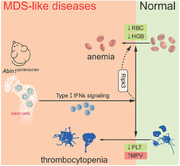

## 1 Introduction

Myelodysplastic syndromes (MDSs) are a group of hematopoietic diseases characterized by myeloid cell dysplasia, ineffective hematopoiesis, and a propensity for bone marrow (BM) failure (BMF) or acute myeloid leukemia.^[^ ^1^ ^]^ Approximately 50–60% of patients with MDSs exhibit chromosomal aberrations, such as chromosome 5q deletion (5q minus (5q–) syndrome or del(5q) MDS), chromosome 7q deletion, chromosome 17p deletion, and other complicated karyotypes.^[^ ^2^ ^]^ Previous studies have shown that several human del(5q) MDS genes, including *TIFAB* (encoding TRAF‐interacting protein with forkhead‐associated domain B), *miR‐146a* (encoding microRNA‐146a), *miR‐145*, and *mDia1* (encoding diaphanous‐related formin‐1), control hematopoiesis by regulating pattern recognition receptor (PRR) signaling.^[^ ^3^ ^]^

As a process vital for normal hematopoiesis, PRR signaling involves several upstream and downstream proteins, such as Toll‐like receptor (TLR), interleukin 1 receptor associated kinase (IRAK) 1, IRAK4, NLR family pyrin domain containing 3 (NLRP3),tumor necrosis factor receptor associated factor (TRAF) 6, S100 calcium binding protein (S100) A8, S100A9, and type I interferons (IFN‐I).^[^ ^4^ ^]^ The production of type I IFNs is regulated through alteration of the axis made up of TRAF3, TANK binding kinase 1 (TBK1), and interferon regulatory factor 3 (IRF3). The recruitment of TBK1 and promotion of type I IFNs are facilitated by the K63‐linked ubiquitination of TRAF3.^[^ ^5^ ^]^ Furthermore, the deubiquitinating enzymes DUBA and tripartite motif containing 24 (TRIM24) can regulate the K63‐linked ubiquitination of TRAF3 to reduce the production of type I IFNs.^[^ ^6^ ^]^ With regard to hematopoiesis, chronic irritation caused by type I IFNs is undesirable for the proliferation and differentiation of hematopoietic stem cells (HSCs),^[^ ^7^ ^]^ indicating an essential role for PRR and type I IFN signaling in the pathogenesis of hematopoietic defects.

*Abin1*, the gene encoding the polyubiquitin‐binding protein A20‐binding inhibitor of nuclear factor‐kappa B activation (ABIN1), resides on chromosome 5q in the region commonly deleted in del(5q) MDS.^[^ ^8^ ^]^ Although the common death of *Abin1^–/–^* mice during embryogenesis could be prevented by deleting tumor necrosis factor receptor superfamily, member 1a (TNFR1), the syndromes associated with post‐implantation development, such as anemia, autoinflammation, and runting disease, were not clearly alleviated.^[^ ^9^ ^]^ Additionally, a previous study reported that the ABIN1‐regulated TLR–myeloid differentiation primary response 88 (MyD88) signaling pathway is responsible for autoinflammation.^[^ ^10^ ^]^ However, the correlation between hematopoiesis and ABIN1 remains unclear.

ABIN1 typically utilizes its ubiquitin‐binding domain (UBD) to regulate the tumor necrosis factor (TNF) and PRR signaling pathways. Once recruited by the TNF‐receptor signaling complex (TNF‐RSC) through methyltransferase 1 (Met1) ubiquitination, ABIN1 recruits the ubiquitin‐editing enzyme TNF alpha induced protein 3 (A20) to control the K63 deubiquitylation of receptor interacting serine/threonine kinase1 (RIPK1) for inducing necroptosis.^[^ ^11^ ^]^ In addition to its contribution to cell death, ABIN1 has been shown to interact with Tax1 binding protein 1 (TAX1BP1) and A20 to form a complex that affects the integration of TRAF3 and TBK1–inhibitor of nuclear factor‐kappa B kinase subunit epsilon (IKKi) through K63 ubiquitination, thereby inhibiting the phosphorylation of IRF3 and secretion of type I IFNs during polyinosinic‐polycytidylic acid (poly(I:C)) transfection or Sendai virus infection.^[^ ^12^ ^]^

Glutamines 477 and 478 are crucial for proper protein interactions in the ABIN1 UBD, recent studies have shown that these glutamines are necessary for K63 ubiquitination, and ABIN1 (Q478) is more conserved than ABIN1 (Q477) across diverse UBDs.^[^ ^13^ ^]^ To better understand the function of the ABIN1 UBD, we generated *Abin1^Q478H/Q478H^* mutant knock‐in mice. In contrast to the embryonic lethality observed in *Abin1^–/–^* mice, the *Abin1^Q478H/Q478H^* mice survived normally but developed MDS‐like diseases, including anemia, thrombocytopenia, and abnormally small megakaryocytes. Furthermore, *Abin1^Q478H/Q478H^* mice exhibited signs of extramedullary hematopoiesis and BMF in the middle and late stages, although only a small proportion of patients with MDSs may experience these symptoms.^[^ ^8^, ^14^ ^]^ The deletion of *Ripk3* caused alleviation of the anemia symptoms in *Abin1^Q478H/Q478H^* mice through necroptosis‐independent mechanisms. More importantly, we found that the co‐deletion of *Ifnar1* (the gene encoding interferon alpha and beta receptor subunit 1) largely relieved the hematopoietic deficiencies in *Abin1^Q478H/Q478H^* mice. These results demonstrate that the ABIN1 (Q478H) mutation represents a unique paradigm in which inhibition of the type I IFN signaling pathway is effective in rescuing *Abin1^Q478H/Q478H^* mice with hematopoietic defects.

## 2 Results

### 2.1 Abin1Q478H/Q478H Mice Developed MDS‐Like Diseases

*Abin1* is located on chromosome 5q in the region commonly deleted in 5q– syndrome, the most distinct of all MDSs.^[^ ^8a^ ^]^ The expression of *Abin1* in mononuclear BM cells of patients with del(5q) MDS is lower than that in patients with non‐del(5q).^[^ ^15^ ^]^ To further determine the correlation between *Abin1* and MDSs, we reanalyzed publicly available datasets of primary BM CD34^+^ cells from patients with del(5q) MDS.^[^ ^16^ ^]^ The *Abin1* mRNA levels were significantly lower in the patient samples than in those from healthy individuals (Figure S1a, Supporting Information). Additionally, we observed that patients with refractory anemia with excess blasts (RAEB)‐type MDSs who had lower levels of ABIN1 expression in their CD34^+^ cells had poorer survival rates (Figure S1b, Supporting Information). These clinical data provide evidence that ABIN1 plays a critical role in the pathogenesis of MDSs. However, the known functions of ABIN1 are currently limited to its role in controlling mouse embryonic development through the regulation of TNF signaling as well as its involvement in the regulation of autoimmunity via TLR–MyD88.^[^ ^9^, ^10^, ^17^ ^]^ Therefore, the specific role of ABIN1 in hematopoiesis remains unclear and requires further investigation.

**Figure 1:**
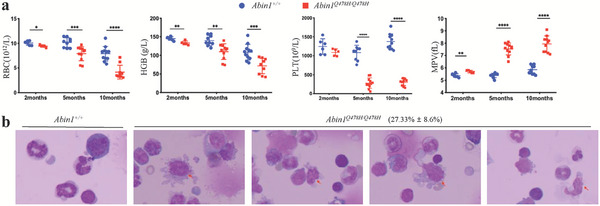
*Abin1^Q478H/Q478H^* mice developed MDS‐like diseases a) Peripheral blood analysis of wild‐type (WT) and *Abin1^Q478H/Q478H^* mice, including red blood cell (RBC), hemoglobin (HGB), and platelet (PLT) levels and mean platelet volume (MPV). (n ≥ 5 mice/group). b) With a magnification of 100×, the Giemsa‐stained bone marrow cytospins from 5‐month‐old *Abin1^Q478H/Q478H^* mice display notably abnormally small megakaryocytes (arrow). (n  ≥ 3 mice/group) The panel data were analyzed using the two‐tailed unpaired Student t‐test, and statistical significance was indicated as follows: **** for P < 0.0001, *** for P < 0.001, ** for P < 0.01, and * for P < 0.05.

ABIN1 contains a UBD that binds to polyubiquitin and polyubiquitinated proteins, as well as a NEMO‐binding domain at the C‐terminus, both of which are involved in the inhibition of antiviral signaling.^[^ ^12^, ^18^ ^]^ Notably, its inhibitory effect against antiviral signaling is reportedly dependent on its binding to polyubiquitin chains, an ability mediated by the UBD domain, as point mutations in this domain can abolish the inhibitory function of the protein.^[^ ^12a^ ^]^ A previous study has shown that ABIN1 (QQ477/478) residues, located in the UBD of ABIN1, are crucial for binding to K63‐linked ubiquitin chains.^[^ ^17^ ^]^ Moreover, ABIN1 (Q478) is evolutionarily more conserved than ABIN1 (Q477) in UBDs across different species.^[^ ^17^ ^]^ To investigate the physiological role of the ubiquitin‐binding ability of ABIN1, we generated *Abin1^Q478H/Q478H^* knock‐in mice expressing the ABIN1 (Q478H) mutation (Figure S1c, Supporting Information). Notably, this mutation did not affect ABIN1 expression (Figure S1d, Supporting Information). Unlike *Abin1^–/–^* mice, which died during embryonic development, *Abin1^Q478H/Q478H^* mice survived normally. No significant differences in body weight, lymph node size, or number of peripheral blood white blood cells (WBCs) were observed between the *Abin1^Q478H/Q478H^* and WT groups (Figure S1e—I, Supporting Information). However, as the mice aged, the *Abin1^Q478H/Q478H^* group showed progressive decreases in circulating red blood cell (RBC) counts and hemoglobin concentrations compared with the age‐matched WT mice (**Figure** **1**a). Additionally, at 5 and 10 months of age, the *Abin1^Q478H/Q478H^* mice exhibited a reduction in platelet count and an increase in mean platelet volume (MPV) in the peripheral blood compared with the age‐matched WT mice (Figure 1a). Notably, these differences were more pronounced in the older mice, indicating that *Abin1^Q478H/Q478H^* mice developed progressive anemia and thrombocytopenia. Moreover, abnormally small megakaryocytes were observed in the BM of *Abin1^Q478H/Q478H^* mice, as assessed using cytospins (Figure 1b). Taken together, these findings suggest that *Abin1^Q478H/Q478H^* mice develop MDS‐like diseases characterized by anemia, thrombocytopenia, and abnormally small megakaryocytes.

### 2.2 Abin1Q478H/Q478H Mice Exhibited Extramedullary Hematopoiesis

**Figure 2:**
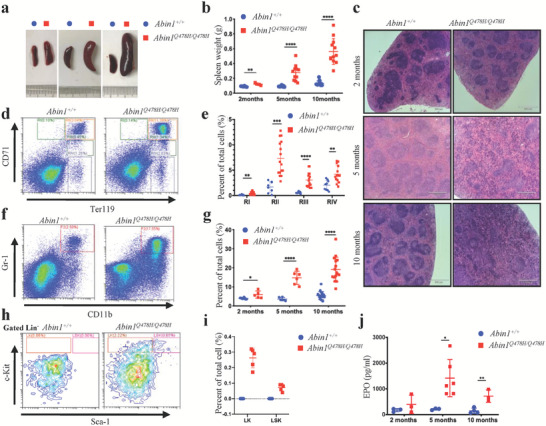
*Abin1^Q478H/Q478H^* mice developed hematopoietic defects. a–b) Different sizes (a) and weights (b) of the spleens of wild‐type (WT) and *Abin1^Q478H/Q478H^* mice at the indicated age. (n ≥ 5 mice/group) c) Hematoxylin and eosin staining of the spleen. Scale bar, 560 µm. Representative of at least three similar‐aged mice at the indicated age per group. d,e) Representative flow cytometry profiles (d) and quantification of the frequencies (e) of the spleen cells of 10‐month‐old WT and *Abin1^Q478H/Q478H^* mice at the indicated erythroid differentiation stages (RI, proerythroblasts; RII, basophilic erythroblasts; RII, chromatophilic erythroblasts; RIV, orthochromatophilic erythroblasts). (n ≥ 5 mice/group) f,g) Representative flow cytometry profiles (f) and quantification of the frequencies (g) of the myeloid (CD11b^+^ and Gr1^+^) cells in the spleens of WT and *Abin1^Q478H/Q478H^* mice at the indicated age. (n  ≥ 5 mice/group) h,i) Representative flow cytometry profiles (h) and quantification of the frequencies (i) of the lineage‐negative‐Kit^+^ (LK) and lineage‐negative‐Sca1^+^‐Kit^+^ (LSK) cells in the spleens of 10‐month‐old WT and *Abin1^Q478H/Q478H^* mice. (n ≥ 5 mice/group) j) Serum erythropoietin (EPO) levels of WT and *Abin1^Q478H/Q478H^* mice at the indicated age. (n ≥ 3 mice/group) The panel data were analyzed using the two‐tailed unpaired Student t‐test, and statistical significance was indicated as follows: **** for P < 0.0001, *** for P < 0.001, ** for P < 0.01, and * for P < 0.05.

In addition to the MDS‐like phenotypes, hematopoietic deficiencies were observed in the *Abin1^Q478H/Q478H^* mice. Specifically, they exhibited splenomegaly, with the increased size of the spleen correlating with a decrease in the number of peripheral blood RBCs (**Figure** **2**a,b). Further evaluation of the spleen histology and cellular composition revealed that the boundary between the white and red pulps appeared more blurred during aging in the *Abin1^Q478H/Q478H^* mice compared with that in the WT mice (Figure 2c). Although there was no significant difference in the frequency of lymphoid cells (CD3^+^ and B220^+^) in the spleen (Figures S1j, Supporting Information), we examined the erythroid and myeloid cells (CD11b^+^ and Gr1^+^) in the *Abin1^Q478H/Q478H^* mouse spleen. For flow cytometric analysis, Ter119 and CD71 antibodies were used to distinguish the cells of the various erythroid differentiation stages, including proerythroblasts, basophilic erythroblasts, chromatophilic erythroblasts, and orthochromatophilic erythroblasts.^[^ ^19^ ^]^ We observed altered erythroid differentiation stages, with increased frequencies of immature erythroid cells (RI, RII, RIII, and RIV) in the *Abin1^Q478H/Q478H^* mouse spleen at both 5 months (Figure S1k, Supporting Information) and 10 months (Figure 2d,e) compared with that in the age‐matched WT mice. Furthermore, the frequency of myeloid cells (CD11b^+^ and Gr1^+^) was significantly higher in the spleens of the *Abin1^Q478H/Q478H^* mice (Figure 2f,g). Additionally, lineage‐negative (Lin^–^) cells in the spleens of the mutant mice at 10 months of age showed a bias toward high Sca‐1 and c‐Kit antigens compared with that observed with the WT mice (Figure 2h,i). These findings suggest that the remarkable splenomegaly in *Abin1^Q478H/Q478H^* mice is due to the expansion of the early erythroid compartment, myeloid cells (CD11b^+^ and Gr1^+^), and HSCs. Previous studies have shown that erythropoietin (EPO) promotes extramedullary hematopoiesis in response to hypoxia and anemia.^[^ ^20^ ^]^ Consistent with this finding, our *Abin1^Q478H/Q478H^* mice also exhibited greatly elevated levels of EPO in their blood plasma compared with the WT mice (Figure 2j). Furthermore, we observed the infiltration of small lymphocytes and granulocytes in the livers of the *Abin1^Q478H/Q478H^* mice (Figure S1l,m, Supporting Information). In conclusion, the data presented here suggest that *Abin1^Q478H/Q478H^* mice display extramedullary hematopoiesis, which may have implications in BM hematological disorders.

### 2.3 The ABIN1 (Q478H) Mutation Induced Bone Marrow Failure Caused By Hematopoietic Stem Progenitor Cell Destruction

**Figure 3:**
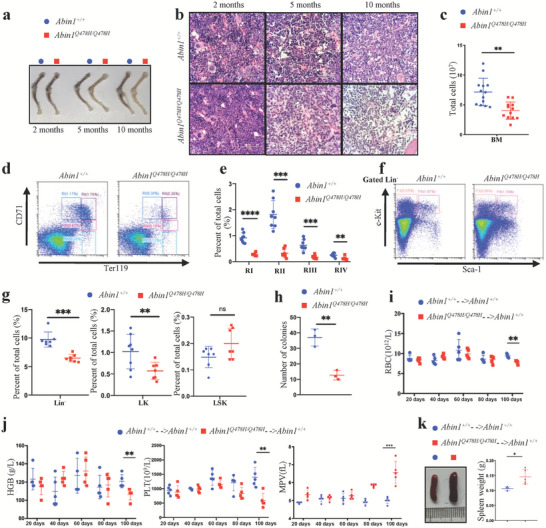
*Abin1^Q478H/Q478H^* mice developed bone marrow failure. a) Femur and tibia of wild‐type (WT) and *Abin1^Q478H/Q478H^* mice at the indicated age. Representative of at least three mice per group. b) Hematoxylin and eosin staining images of bone marrow (BM). Representative of at least three mice at the indicated age per group. c) Reduced total BM cells in 5‐month‐old WT and *Abin1^Q478H/Q478H^* mice. BM cells from 2 femurs and 2 tibias were harvested from 5‐month‐old WT and *Abin1^Q478H/Q478H^* mice. (n ≥ 12 mice/group) d,e) Representative flow cytometry profiles (d) and quantification of the frequencies (e) of the BM cells of 5‐month‐old WT and *Abin1^Q478H/Q478H^* mice at the indicated erythroid differentiation stages (RI, proerythroblasts; RII, basophilic erythroblasts; RII, chromatophilic erythroblasts; RIV, orthochromatophilic erythroblasts). (n ≥ 7 mice/group) f) Representation flow cytometry profiles of lineage‐negative‐Kit^+^ (LK) and lineage‐negative‐Sca1^+^‐Kit^+^ (LSK) BM cells of 5‐month‐old WT and *Abin1^Q478H/Q478H^* mice. g) Quantification of the frequencies of Lin^–^, LK, and LSK BM cells of the 5‐month‐old WT and *Abin1^Q478H/Q478H^* mice. (n ≥ 7 mice/group) h) Quantification of the total colony number in colony‐forming unit (CFU) assays. LSK (1 × 10^3^) cells isolated from 5‐month‐old WT and *Abin1^Q478H/Q478H^* BM cells were plated for each assay. (n ≥ 3 mice/group) I,j) Peripheral blood analysis of the 2‐month‐old WT and *Abin1^Q478H/Q478H^* BM cell‐recipient mice, including red blood cell (RBC) count (i), hemoglobin (HGB) level, platelet (PLT) count, and mean platelet volume (MPV) (j). (n ≥ 4 mice/group) k) Different sizes and weights of the spleens of the 2‐month‐old WT and *Abin1^Q478H/Q478H^* BM cell‐recipient mice at 100 days after cell transplantation (n ≥ 4 mice/group) The panel data were analyzed using the two‐tailed unpaired Student t‐test, and statistical significance was indicated as follows: **** for P < 0.0001, *** for P < 0.001, ** for P < 0.01, and * for P < 0.05.

Previous studies have reported that individuals with del(5q) MDS are at high risk of developing BMF.^[^ ^21^ ^]^ Therefore, we investigated whether the *Abin1^Q478H/Q478H^* mice developed MDS‐mediated BMF. As expected, the color of the femur and tibia of the mutant mice varied from red to white (**Figure** **3**a), which was consistent with the hematoxylin and eosin staining results that showed reduced BM cell density in these mice at both 5 and 10 months of age (Figure 3b). Moreover, reticular fiber staining revealed severe myelofibrosis in the BM of *Abin1^Q478H/Q478H^* mice compared with that in WT mice (Figure S2a, Supporting Information). At 5 months of age, the mutant mice showed a reduction in the total number of BM cells (Figure 3c). Furthermore, all cells of the various erythroid differentiation stages, including RI, RII, RIII, and RIV, were present in lower numbers than those in the WT mice (Figure 3d,e). To determine whether the decreased erythroid cell number was due to failure of the progenitor cells to differentiate in the BM, we analyzed the hematopoietic stem progenitor cell (HSPC) in the BM (Figure 3f). Although the frequency of Lin^–^‐Sca1^+^‐c‐Kit^+^ (LSK) cells was slightly above normal, that of *Abin1^Q478H/Q478H^* Lin^–^ and Lin^–^‐c‐Kit^+^ (LK) cells was significantly decreased (Figure 3g), and the frequencies of megakaryocyte‐erythrocyte progenitors (MEPs), granulocyte‐macrophage progenitors (GMPs), and common myeloid progenitors (CMPs) also showed a decreasing trend compared with those of the WT mice (Figure S2b, Supporting Information). Since the overall proportion of LSK cells did not change significantly, it is important to perform cell sorting and cell number analysis for LSK cells. Therefore, we conducted further experiments on LSK cells. Flow cytometry results revealed that the proportion of hematopoietic progenitor cells‐2 (HPC‐2) cells was upregulated in both 5‐month‐old and 9‐month‐old *Abin1^Q478H/Q478H^* mice compared with age‐matched WT mice. Specifically, in 5‐month‐old *Abin1^Q478H/Q478H^* mice, we observed an increase in the number of HPC‐2 cells, accompanied by a decrease in the number of multipotent hematopoietic potential (MPP) and multipotent long‐term HSCs (LT‐HSC) cells compared with age‐matched WT mice. In 9‐month‐old *Abin1^Q478H/Q478H^* mice, there was a decrease in the proportion of MPP cells, accompanied by a reduction in the number of hematopoietic progenitor cells‐1(HPC‐1) cells, and LT‐LSK cells compared with age‐matched WT mice. These results suggest that *Abin1^Q478H/Q478H^* mice exhibit abnormal proportions and numbers of LSK cell subtypes (Figure S2c,d, Supporting Information). Furthermore, Colony‐Forming Units (CFU) assays showed a reduced self‐renewal and proliferative capacity of total LSK cells in 5‐month‐old *Abin^Q478H/Q478H^* mice (Figure 3h). To investigate the mechanism underlying hematopoiesis defeat, we further conducted CFU assays on subpopulations of HSPC in *Abin^Q478H/Q478H^* mice targeting HPC‐1, HPC‐2, MPP, and LT‐HSC. The results showed a lower number of cell colonies formed by HPC‐1, HPC‐2, MPP, and LT‐HSC cells in 5‐month‐old *Abin^Q478H/Q478H^* mice compared with age‐matched WT mice (Figure S2e, Supporting Information). Consistently, the cell numbers undergoing CFU assays division of HPC‐1, HPC‐2, MPP, and LT‐HSC were also lower in *Abin^Q478H/Q478H^* mice compared with WT mice (Figure S2e, Supporting Information). Additionally, the cobblestone‐area forming cell (CAFC) assays also revealed a decrease in the number of cell colonies formed by HPC‐1, HPC‐2, MPP, and LT‐HSC cells in *Abin^Q478H/Q478H^* mice, with MPP and LT‐HSC cells forming smaller colonies compared with WT cells (Figure S2f,g, Supporting Information). Taken together, these results suggest that the ABIN1(Q478H) mutation results in impaired HSPC proliferation and differentiation.

Next, we determined whether the hematopoietic deficiencies observed in *Abin1^Q478H/Q478H^* cells were derived from the BM stroma or BM cells. WT and *Abin1^Q478H/Q478H^* BM cells were transplanted into lethally irradiated mice. At 100 days post‐transplantation, mice that received transplants of *Abin1^Q478H/Q478H^* BM cells exhibited anemia with decreased red blood cell (RBC) counts and hemoglobin levels (Figure 3i,j), as well as thrombocytopenia with reduced platelet counts and abnormal mean platelet volume (MPV) in the peripheral blood (Figure 3j), compared with mice that received WT BM cells. Moreover, the mice that received *Abin1^Q478H/Q478H^* BM cells displayed spleen enlargement (Figure 3k), and an increased frequency of erythropoiesis and myeloid cells (CD11b^+^ and Gr1^+^) (Figure S2h,I, Supporting Information) compared with the mice that received WT BM cells. Overall, these findings suggest that the BM cells of *Abin1^Q478H/Q478H^* mice are unable to produce a sufficient amount of healthy blood cells, resulting in the development of MDS‐like diseases.

### 2.4 RIPK3 Controlled Abin1Q478H/Q478H Anemia through Necroptosis‐Independent Function

**Figure 4:**
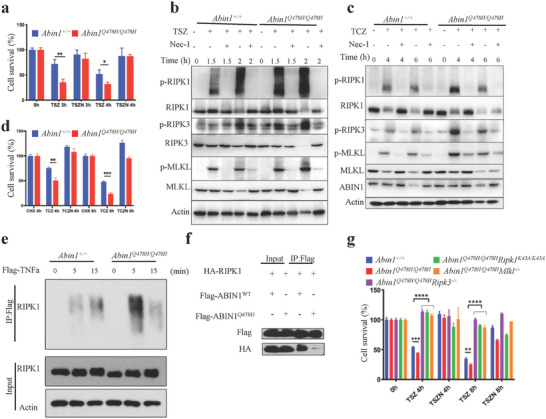
*Abin1^Q478H/Q478H^* was sensitive to necroptosis a,b) Cell survival (a) and western blot assays (b) of wild‐type (WT) and *Abin1^Q478H/Q478H^* primary mouse dermal fibroblasts (MDFs) treated with TNFα/Smac/zVAD (TSZ) in the presence or absence of Nec‐1 for the indicated periods. c,d) Cell survival (d) and western blot assays (c) of WT and *Abin1^Q478H/Q478H^* primary MDFs treated with TNFα/cycloheximide/zVAD (TCZ) in the presence or absence of Nec‐1s for the indicated periods. e) WT and *Abin1^Q478H/Q478H^* primary MDFs were treated with Flag‐TNFα (100 ng ml^‐1^) for the indicated times, the TNF‐receptor signaling complex (TNF‐RSC) was pulled down by Flag‐beads in primary MDFs using the indicated genotypes, and RIPK1 ubiquitination was detected using western blotting. f) Immunoblot analysis of ABIN1‐RIPK1 and ABIN1(Q478H)‐RIPK1 interaction in HEK293T cells transfected with HA‐RIPK1 and Flag‐ABIN1 or Flag‐ABIN1 (Q478H) plasmid. g) Survival of WT, *Abin1^Q478H/Q478H^*, *Abin1^Q478H/Q478H^Ripk3^–/–^*, *Abin1^Q478H/Q478H^Ripk1^K45A/K45A^*, and *Abin1^Q478H/Q478H^ Mlkl^–/–^* primary MDFs treated with TSZ in the presence or absence of Nec‐1s for the indicated periods. T (TNFα, 20 ng ml^−1^), S (Smac, 1 µM), C (CHX, 40 µg ml^−1^), Z (zVAD, 20 µM), and N (Nec‐1, 30 µM). The panel data were analyzed using the two‐tailed unpaired Student t‐test, and statistical significance was indicated as follows: **** for P < 0.0001, *** for P < 0.001, ** for P < 0.01, and * for P < 0.05.

Both *Abin1* deficiency and *Abin1^UBD/UBD^* mutation render cells sensitive to necroptosis,^[^ ^11^, ^17^ ^]^ suggesting that the *Abin1^Q478H/Q478H^* mutation, which results in MDS‐like diseases in the mice, may be functionally associated with necroptotic cell death. Therefore, we investigated the effect of the ABIN1 (Q478H) mutation on necroptosis. As expected, when necroptosis was induced in MDFs using TNFα plus Smac mimetics (Smac) and the pan‐caspase inhibitor zVAD‐FMK (zVAD), the luminescent cell viability assay results revealed a lower percentage of MDF survival in the *Abin1^Q478H/Q478H^* group than in the WT group (**Figure** **4**a). The hallmark of necroptosis is phosphorylation of the RIPK1–RIPK3–mixed lineage kinase domain like pseudokinase (MLKL) axis, which can be rescued by Nec‐1, a necroptosis kinase inhibitor targeting RIPK1.^[^ ^22^ ^]^ We evaluated the necroptosis process upon TNFα/Smac/zVAD (TSZ) stimulation and found that the induction of RIPK1–RIPK3–MLKL axis phosphorylation was elevated in *Abin1^Q478H/Q478H^* cells but could be blocked by Nec‐1 (Figure 4b). Similarly, *Abin1^Q478H/Q478H^* MDFs were sensitive to TNFα/cycloheximide (CHX)/zVAD‐induced necroptosis, displaying a lower cell survival percentage (Figure 4d), and higher levels of p‐RIPK1, p‐RIPK3, and p‐MLKL were detected in the *Abin1^Q478H/Q478H^* MDFs than in the WT MDFs upon TNFα/CHX/zVAD (TCZ) stimulation (Figure 4c). We also treated bone marrow‐derived macrophages (BMDMs) with TNF‐α plus Smac and zVAD or LPS plus zVAD to induce necroptosis. Notably, we observed that *Abin1^Q478H/Q478H^* BMDMs exhibited excessive necroptosis compared with WT BMDMs, which was significantly suppressed by RIPK1 kinase inhibitor Nec‐1(Figure S3a, Supporting Information). A previous study proposed that ABIN1 modulates the ubiquitination of RIPK1 to regulate necroptosis.^[^ ^11a^ ^]^ Therefore, we used Flag‐TNFα to pull down the TNF‐RSC and found that *Abin1^Q478H/Q478H^* MDFs exhibited elevated levels of RIPK1 ubiquitination (Figure 4e). Furthermore, the mutant ABIN1 (Q478H) protein showed a lower ability than WT ABIN1 to bind to RIPK1 (Figure 4f). These results suggest that the ABIN1 (Q478H) mutation promotes RIPK3‐, MLKL‐, and RIPK1 kinase‐mediated necroptosis by regulating the ubiquitination of RIPK1.

**Figure 5:**
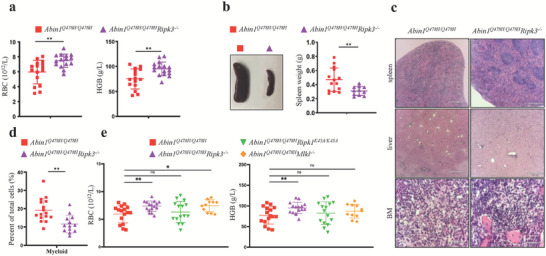
*RIPK3^–/–^* alleviated anemia in *Abin1^Q478H/Q478H^* mice via necroptosis‐independent function a) Peripheral blood analysis of 10‐month‐old *Abin1^Q478H/Q478H^* and *Abin1^Q478H/Q478H^Ripk3^–/–^* mice, including red blood cell (RBC) counts and hemoglobin (HGB) levels. (n ≥ 16 mice/group). b) Different sizes and weights of the spleens of the 10‐month‐old *Abin1^Q478H/Q478H^* and *Abin1^Q478H/Q478H^Ripk3^–/–^* mice. (n ≥ 12 mice/group) c) Hematoxylin and eosin staining of spleen, liver, and bone marrow samples. Representative of at least three 10‐month‐old mice per group. d) Quantification of the myeloid (CD11b^+^ and Gr1^+^) frequencies of the 10‐month‐old *Abin1^Q478H/Q478H^* and *Abin1^Q478H/Q478H^Ripk3^–/–^* mice. (n ≥ 13 mice/group) e) Peripheral blood analysis of 10‐month‐old wild‐type (WT), *Abin1^Q478H/Q478H^*, *Abin1^Q478H/Q478H^Ripk3^–/–^*, *Abin1^Q478H/Q478H^Ripk1^K45A/K45A^*, and *Abin1^Q478H/Q478H^ Mlkl^–/–^* mice, including red blood cell (RBC) counts and hemoglobin (HGB) levels. (n ≥ 11 mice/group) The panel data were analyzed using the two‐tailed unpaired Student t‐test, and statistical significance was indicated as follows: **** for P < 0.0001, *** for P < 0.001, ** for P < 0.01, and * for P < 0.05.

Considering that RIPK3 deletion could prevent compensated anemia in glutathione peroxidase 4 (Gpx4)‐deficient mice and BMF in *Tak1^mut^Tnfr^−/−^* mice,^[^ ^23^ ^]^ we investigated whether *Abin1^Q478H/Q478H^* drives hematopoiesis defects through an RIPK3‐dependent pathway. By crossing RIPK3‐knockout mice with *Abin1^Q478H/Q478H^* mice, we generated *Abin1^Q478H/Q478H^Ripk3^–/–^* mice, which displayed increased RBC and hemoglobin levels (**Figure** **5**a) as well as alleviated splenomegaly compared with *Abin1^Q478H/Q478H^* mice (Figure 5b). Furthermore, hematoxylin and eosin staining showed that *Abin1^Q478H/Q478H^Ripk3^–/–^* mice had a more normal organ architecture than WT mice, with distinctive red and white pulps in the spleen and lower infiltration of small lymphocytes and granulocytes in the liver (Figure 5c). Although the frequencies of the different erythroid developmental stages did not differ significantly (Figure S3b, Supporting Information), the *Abin1^Q478H/Q478H^Ripk3^–/–^* mice had decreased frequencies of myeloid cells (CD11b^+^ and Gr1^+^) in the spleen compared with the *Abin1^Q478H/Q478H^* mice (Figure 5d). Moreover, our findings also showed that 10‐month‐old *Abin1^Q478H/Q478H^Ripk3^–/–^* mice had a remarkably higher density of BM cells and a visibly deeper red coloration of the BM than *Abin1^Q478H/Q478H^* mice (Figure 5c; Figure S3c, Supporting Information). Taken together, these results indicate that *Ripk3* deletion can partially ameliorate anemia defects in *Abin1^Q478H/Q478H^* mice.

Given that RIPK3 participates in various necroptosis‐independent signaling pathways, such as apoptosis, senescence, inflammation, and autophagy,^[^ ^24^ ^]^ we further examined whether its deletion prevents anemia in *Abin1^Q478H/Q478H^* mice by blocking RIPK1–RIPK3–MLKL axis‐mediated necroptosis. We crossed RIPK1 kinase‐dead mice (*Ripk1^K45A/K45A^*) and *Mlkl*‐knockout mice with *Abin1^Q478H/Q478H^* mice. First, we examined the roles of the MLKL and RIPK1 kinase‐dead mutations in necroptosis in *Abin1^Q478H/Q478H^* MDFs and found that the TSZ‐stimulated *Abin1^Q478H/Q478H^* cells exhibited increased necroptosis that was dependent on RIPK1 kinase, RIPK3, and MLKL (Figure 4g). However, the RBC count and hemoglobin level in *Abin1^Q478H/Q478H^Ripk1^K45A/K45A^* mice did not show any increase compared with those in *Abin1^Q478H/Q478H^* mice (Figure 5e). Furthermore, although a slight difference in the RBC count was observed, there were no significant difference in hemoglobin levels between the *Abin1^Q478H/Q478H^Mlkl^–/–^* and *Abin1^Q478H/Q478H^* mice. Moreover, no significant difference in spleen weight was observed among the *Abin1^Q478H/Q478H^*, *Abin1^Q478H/Q478H^Ripk1^K45A/K45A^*, and *Abin1^Q478H/Q478H^Mlkl^–/–^* mice (Figure S3d, Supporting Information). These results showed that the deletion of RIPK3, but not the MLKL deficiency or RIPK1 kinase‐dead mutation, could rescue *Abin1^Q478H/Q478H^* mice from anemia, suggesting that RIPK3 promotes anemia in *Abin1^Q478H/Q478H^* mice through necroptosis‐independent functions.

Notably, although anemia in *Abin1^Q478H/Q478H^* mice was ameliorated by *Ripk3* deficiency, the *Abin1^Q478H/Q478H^Ripk3^–/–^*, *Abin1^Q478H/Q478H^Ripk1^K45A/K45A^*, and *Abin1^Q478H/Q478H^Mlkl^–/–^* mice still showed severe thrombocytopenia, similar to the *Abin1^Q478H/Q478H^* mice (Figure S3e,f, Supporting Information). This suggests that the thrombocytopenia observed in *Abin1^Q478H/Q478H^* mice is not correlated with RIPK3 or necroptosis. Taken together, these data suggest that the deletion of *Ripk3* could partially ameliorate MDS‐like diseases in *Abin1^Q478H/Q478H^* mice in a necroptosis‐independent manner.

### 2.5 The Upregulation of Type I Interferons was Responsible for The Hematopoietic Deficiencies in Abin1Q478H/Q478H Mice

**Figure 6:**
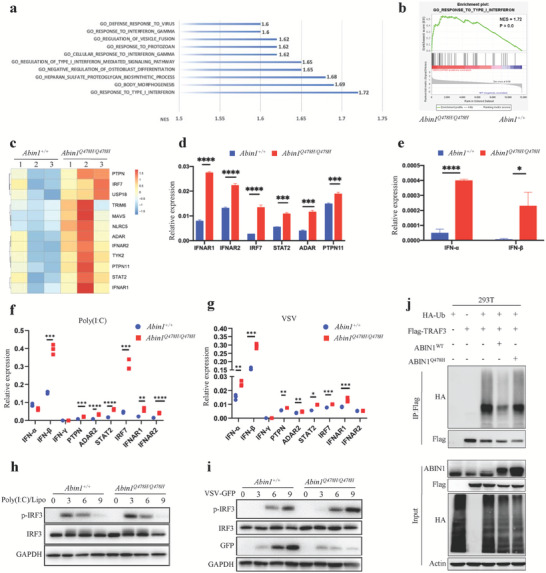
Enhanced expression of type I interferons in *Abin1^Q478H/ Q478H^* bone marrow cells. a) Gene set enrichment analysis (GSEA) of enriched Gene Ontology (GO) terms for biological processes in lineage‐negative (Lin^–^) bone marrow (BM) cells from 5‐month‐old wild‐type (WT) and *Abin1^Q478H/Q478H^* mice. b) GO terms of the “response to type I interferon” in Lin^–^ BM cells from 5‐month‐old WT and *Abin1^Q478H/Q478H^* mice. c) Differentially expressed genes related to type I interferon (IFN) were identified through expression profiling of Lin^–^ BM cells from 5‐month‐old WT and *Abin1^Q478H/Q478H^* mice. d) qPCR analysis of representative type I interferon‐related genes in Lin^–^ BM cells from 5‐month‐old WT and *Abin1^Q478H/Q478H^* mice. e) qPCR analysis of IFNα and IFNβ in Lin^–^ BM cells from 5‐month‐old WT and *Abin1^Q478H/Q478H^* mice. f) Expression of type I IFN‐related genes was assessed by qPCR in bone marrow‐derived macrophages (BMDMs) from WT and *Abin1^Q478H/Q478H^* mice transfected with poly(I:C) for 4 h. g) Immunoblot analysis of phosphorylated and total IRF3 in whole‐cell lysates of WT and *Abin1^Q478H/Q478H^* BMDMs transfected with poly(I: C) at the indicated time points. h) qPCR‐assessed expression of type I IFN‐related genes in BMDMs from WT and *Abin1^Q478H/Q478H^* mice infected with vesicular stomatitis virus (VSV) for 4 h. i) Immunoblot analysis of phosphorylated and total IRF3 in whole‐cell lysates of WT and *Abin1^Q478H/Q478H^* BMDMs infected with VSV at the indicated time points. j) TRAF3 ubiquitination in 293T cells transfected with the indicated expression vectors, as assessed by immunoblot analysis with anti‐HA after immunoprecipitation with anti‐FLAG or by immunoblot analysis with input proteins in lysates without immunoprecipitation. The panel data were analyzed using the two‐tailed unpaired Student t‐test, and statistical significance was indicated as follows: **** for P < 0.0001, *** for P < 0.001, ** for P < 0.01, and * for P < 0.05.

To investigate the mechanism underlying the pathogenesis of hematopoietic deficiencies in *Abin1^Q478H/Q478H^* mice, we conducted RNA‐seq analysis of Lin^–^ BM cells from 5‐month‐old mice to gain a global view of the transcriptome profile. Gene set enrichment analysis (GSEA) revealed that multiple Gene Ontology terms in the Biological Processes category were significantly upregulated in the *Abin1^Q478H/Q478H^* group, particularly those related to the reaction to viruses, including “response to type I interferon,” “regulation of type I IFNs‐mediated signaling pathway,” and “viral genome replication” gene sets (**Figure** **6**a). Notably, the “response to type I interferon” gene set was significantly enriched in the transcriptome of *Abin1^Q478H/Q478H^* Lin^–^ cells (Figure 6b). Furthermore, our analysis of gene ontology related to interferon revealed that the expression levels of interferon‐related gene sets were consistently elevated in *Abin^Q478H/Q478H^* compared with WT HSPC (Figure S4d, Supporting Information). Selected altered type I IFN‐associated genes (Figure 6c) were validated using qPCR (Figure 6d), including *ADAR* (encoding adenosine deaminase RNA specific) and *PTPN11* (encoding protein tyrosine phosphatase non‐receptor type 11), which are essential for RBC and platelet production, respectively.^[^ ^7a^ ^25^ ^]^ These findings provide evidence that Lin^–^ BM cells from *Abin1^Q478H/Q478H^* mice express remarkably higher levels of type I IFNs than Lin^–^ BM cells from WT mice do, as confirmed by qPCR analysis (Figure 6e). Although the cellular composition of Lin^–^ cells differs between *Abin1^Q478H/Q478H^* and WT mice, the RNA analysis suggested a potential correlation between high IFN expression and the observed hematopoietic deficiencies in *Abin1^Q478H/Q478H^* mice.

Previous studies have reported that *Abin1^–/–^* macrophages are sensitive to RNA virus‐induced PRR signal activation, which regulates type I IFNs production.^[^ ^12a^ ^]^ To further investigate the effects of the ABIN1 (Q478H) mutation on IFN production, we transfected synthetic poly(I:C) into BM‐derived macrophages (BMDMs) to simulate RNA virus infection. Remarkably, *Abin1^Q478H/Q478H^* BMDMs exhibited elevated expression of *Ifn‐β* and other genes associated with type I IFNs. These findings were consistent with the stronger activation of IRF3 observed under the same poly(I: C)‐stimulated conditions (Figure 6f,g). Next, we investigated whether the ABIN1 (Q478H) mutation causes resistance to RNA virus infection. To study this, we infected BMDMs with vesicular stomatitis virus G protein tagged with green fluorescent protein (VSV‐GFP). The *Abin1^Q478H/Q478H^* BMDMs exhibited remarkably increased expression of type I IFNs (IFN‐β, IFN‐α) and downstream type I IFN genes upon VSV‐GFP infection (Figure 6h). Notably, VSV propagation was significantly lower in *Abin1^Q478H/Q478H^* BMDMs, possibly because of the higher activation of IRF3 observed in these cells compared with that in WT BMDMs (Figure 6i). Given the fact that TRAF3 undergoes different types of ubiquitination by distinct E3 ubiquitin ligases during RNA virus infection, and the ABIN1‐bound A20 protein disrupts the TRAF3–TBK1–IKKi signaling module,^[^ ^5^, ^6^, ^12^ ^]^ we hypothesized that ABIN1 functions as a regulator of TRAF3 in the regulation of type I IFN gene expression. Our findings indicated that ABIN1 overexpression greatly reduced the ubiquitination of TRAF3, whereas ABIN1 (Q478H) overexpression did so only slightly (Figure 6j). Overall, our results indicated that ABIN1 (Q478) plays a crucial role in regulating TRAF3 ubiquitination, which is necessary for the appropriate expression of type I IFNs.

**Figure 7:**
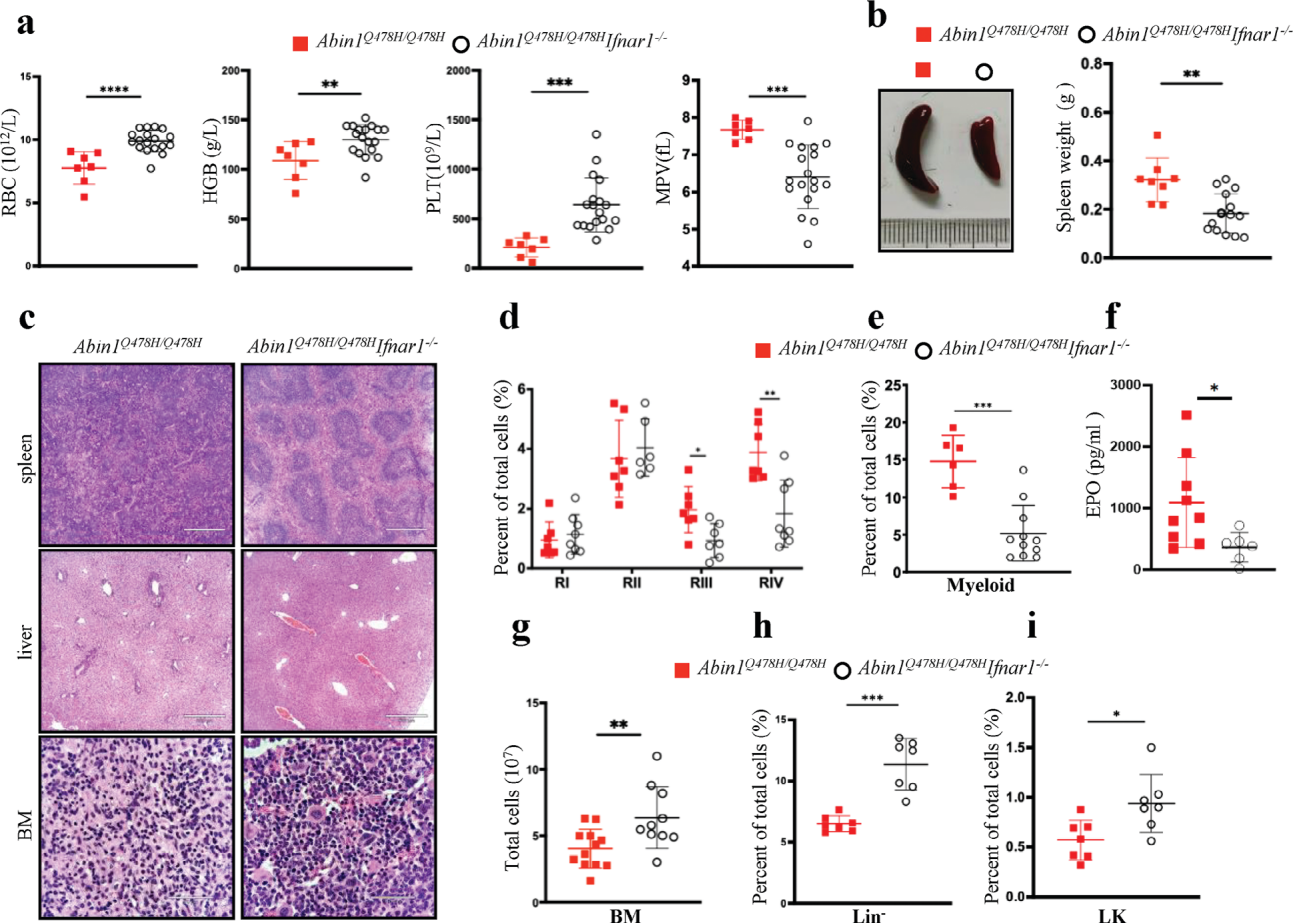
High‐level expression of type I interferons contributed to hematopoietic deficiencies development in *Abin1^Q478H/Q478H^* mice. a) Peripheral blood analysis of 5‐month‐old *Abin1^Q478H/Q478H^* and *Abin1^Q478H/Q478H^Ifnar1^–/–^* mice, including red blood cell (RBC) count, hemoglobin (HGB) level, platelet (PLT) count, and mean platelet volume (MPV). (n ≥ 7 mice/group) b) Different size and weight of the spleens of 5‐month‐old *Abin1^Q478H/Q478H^* and *Abin1^Q478H/Q478H^Ifnar1^–/–^* mice. c) Hematoxylin and eosin staining of spleen, liver, and bone marrow (BM) samples. Representative of at least three 5‐month‐old mice per group. (n ≥ 8 mice/group) d) Quantification of the frequencies of the spleen cells of the 5‐month‐old *Abin1^Q478H/Q478H^* and *Abin1^Q478H/Q478H^Ifnar1^–/–^* mice at the indicated erythroid differentiation stages (RI, proerythroblasts; RII, basophilic erythroblasts; RII, chromatophilic erythroblasts; RIV, orthochromatophilic erythroblasts). (n  ≥ 6 mice/group) e) Quantification of the myeloid (CD11b^+^ and Gr1^+^) frequencies of the 5‐month‐old *Abin1^Q478H/Q478H^* and *Abin1^Q478H/Q478H^Ifnar1^–/–^* mouse spleens. (n  ≥ 6 mice/group) f) Serum erythropoietin (EPO) level in 5‐month‐old *Abin1^Q478H/Q478H^* and *Abin1^Q478H/Q478H^Ifnar1^–/–^* mice. (n ≥ 6 mice/group) g) Total BM cells from 2 femurs and 2 tibias were harvested from 5‐month‐old *Abin1^Q478H/Q478H^* and *Abin1^Q478H/Q478H^Ifnar1^–/–^* mice. (n ≥ 10 mice/group) h) Quantification of the frequencies of lineage‐negative (Lin^–^) BM cells in the 5‐month‐old *Abin1^Q478H/Q478H^* and *Abin1^Q478H/Q478H^Ifnar1^–/–^* mice. (n ≥ 7 mice/group) i) Quantification of the frequencies of the LK BM cells in the 5‐month‐old *Abin1^Q478H/Q478H^* and *Abin1^Q478H/Q478H^Ifnar1^–/–^* mice. (n ≥ 7 mice/group) The panel data were analyzed using the two‐tailed unpaired Student t‐test, and statistical significance was indicated as follows: **** for P < 0.0001, *** for P < 0.001, ** for P < 0.01, and * for P < 0.05.

Previous studies have indicated that chronic activation of the IFN‐α pathway can impair the hematopoietic function of HSCs. However, *Ifnar1^–/–^* mice have been shown to prevent HSC exhaustion resulting from chronic stimulation with type I IFNs.^[^ ^7^ ^]^ To further investigate whether *Abin1^Q478H/Q478H^* mice develop hematopoietic diseases as a result of the excessive activation of type I IFN signaling, we crossed *Ifnar1*‐knockout mice with *Abin1^Q478H/Q478H^* mice. Although dysplastic megakaryocytes could still be detected in the BM of *Abin1^Q478H/Q478H^Ifnar1^–/–^* mice, 5‐month‐old mice, and 9‐month‐old of this mutant strain displayed remarkably higher counts of circulating RBCs and platelets than did *Abin1^Q478H/Q478H^* mice (**Figure** **7**a; Figure S4b, Supporting Information). Furthermore, these mice showed marked improvement in their hemoglobin concentration and a reduction in their MPV compared with the *Abin1^Q478H/Q478H^* mice (Figure 7a; Figure S4b, Supporting Information). These findings suggest that *Abin1^Q478H/Q478H^Ifnar1^–/–^* mice experienced relief from anemia and thrombocytopenia compared with *Abin1^Q478H/Q478H^* mice.

Regarding extramedullary hematopoiesis, the spleens of the *Abin1^Q478H/Q478H^Ifnar1^–/–^* mice appeared to be smaller, and the boundary between the white and red pulps was more distinct than that of the *Abin1^Q478H/Q478H^* mice (Figure 7b,c). Furthermore, the livers of *Abin1^Q478H/Q478H^Ifnar1^–/–^* mice did not exhibit infiltration by small lymphocytes and granulocytes (Figure 7c). Additionally, the spleens of *Abin1^Q478H/Q478H^Ifnar1^–/–^* mice showed a much lower frequency of altered erythroid differentiation stages of immature erythroid cells (identified as RIII and RIV) and myeloid cells (CD11b^+^ and Gr1^+^) compared with those of *Abin1^Q478H/Q478H^* mice (Figure 7d,e). EPO levels were significantly lower in the *Abin1^Q478H/Q478H^Ifnar1^–/–^* mice than in the *Abin1^Q478H/Q478H^* mice (Figure 7f). These results suggest that *Ifnar1* deficiency alleviates extramedullary hematopoiesis in *Abin1^Q478H/Q478H^* mice. We found that the reduced number of total BM cells, Lin^–^ BM cells, and LK BM cells in *Abin1^Q478H/Q478H^* mice could also be restored through *Ifnar1* knockout (Figure 7g,i). Thus, these results indicate that *Ifnar1* knockout alleviates the development of hematopoietic deficiencies in *Abin1^Q478H/Q478H^* mice. Taken together, our data suggest that *Abin1^Q478H/Q478H^* mice spontaneously develop hematopoietic deficiencies because of the elevated expression of type I IFNs.

## 3 Discussion

Aside from showing a decrease in hemoglobin concentration, *Abin1^–/–^* mice are also susceptible to embryonic lethality, which prevents further investigation of the role of ABIN1 in hematopoietic development.^[^ ^9^ ^]^ Therefore, the relationship between hematopoiesis and ABIN1 expression remains unclear. In this study, we generated *Abin1^Q478H/Q478H^* knock‐in mice with a mutation that disrupts the polyubiquitin‐binding site. These mice exhibited anemia, thrombocytopenia, abnormally small megakaryocytes, extramedullary hematopoiesis, and BMF, indicating that the polyubiquitin‐binding function of ABIN1 through its UBD is crucial for normal hematopoietic development. *Ripk3* deficiency could alleviate anemia and splenomegaly—but not thrombocytopenia—in a necroptosis‐independent manner in *Abin1^Q478H/Q478H^* mice. However, the co‐deletion of *Ifnar*1, which blocks type I IFN signaling, greatly ameliorated anemia, thrombocytopenia, and extramedullary hematopoiesis in these mutant mice. These results suggest that ABIN1(Q478) prevents hematopoietic deficiencies by regulating the expression of type I IFNs (Figure S5, Supporting Information). Therefore, the beneficial effect of *Ifnar*1 deletion in *Abin1^Q478H/Q478H^* mice serves as a promising novel strategy for treating hematopoietic deficiencies and may offer new opportunities for therapeutic interventions in patients suffering from hematopoietic diseases, including del(5q) MDS.

A previous study suggested that the UBD of ABIN1 was important for controlling the TNF signaling and TLR–MyD88 pathways.^[^ ^9^, ^10^, ^11^ ^]^ In this study, we found that *Abin1^Q478H/Q478H^* knock‐in mice developed spontaneous anemia and thrombocytopenia through excessive activation of TRAF3‐regulated type I IFN signaling. This suggests that ABIN1 can bind to the TNF‐RSC, MyD88, and TRAF3 complexes through its UBD, thereby regulating the innate immunity signaling pathway during embryonic development, inflammation, and hematopoiesis. Furthermore, the observation that *Abin1^–/–^* mice develop both autoimmune disorders and anemia could potentially provide a molecular explanation for the high risk of MDSs associated with autoimmune disorders in clinical research.^[^ ^26^ ^]^

*Abin1^Q478H/Q478H^* mice and mice with ABIN1 UBD domain deletions display significantly distinct phenotypes. Mice with ABIN1 UBD domain deletion were embryonic lethality, like ABIN1 knock‐out mice, both of which can be rescued through TNFR1 knock‐out and RIPK1(K45A) mutation.^[^ ^11^ ^]^ In contrast, *Abin1^Q478H/Q478H^* mutant mice are viable but exhibit phenotypes reminiscent of myelodysplastic syndrome (MDS). Given that the ABIN1 (Q478H) mutation is located within the ABIN1 UBD domain, which is primarily responsible for ubiquitin binding, we speculated that the observed difference in the mice phenotypes might be attributed to the potential distinct impacts of the ABIN1 (Q478H) mutation and ABIN1 UBD domain deletion on the capacity of ubiquitin binding.

Previous studies have demonstrated that type I IFNs play a role in the collapse of HSPCs during infection or sterile inflammation.^[^ ^27^ ^]^ However, the relationship between MDSs and type I IFN signaling remains unclear. By using the *Abin1^Q478H/Q478H^* mouse model for MDS‐like diseases, we have provided evidence that dysregulation of PRR signaling can affect the production of type I IFNs, leading to hematopoietic defects. In humans, several genes associated with del(5q) MDS, such as *TIFAB*, *miR‐145*, *miR‐146a*, and *mDia1*,^[^ ^3^, ^28^ ^]^ have been shown to protect mice from hematopoietic dysfunction via PRR signaling. Studies have shown that PRR signaling induces type I IFN production via the TRAF3–TBK1–IRF3 pathway.^[^ ^5^, ^29^ ^]^ Recent studies have also revealed that type I IFNs are responsible for defective hematopoiesis resulting from Plk1‐interacting checkpoint helicase (PICH) deficiency.^[^ ^30^ ^]^ Although clinical reports of patients with ABIN1 (Q478) mutation‐carrying MDSs are currently unavailable, a recent case study reported a patient with MDS with a ABIN1::platelet‐derived growth factor receptor beta (PDGFRB) rearrangement.^[^ ^31^ ^]^ This genetic rearrangement results in the deletion of 12–18 ABIN1 exons, which are located within the region encoding the ABIN1 UBD and encompass the ABIN1 (Q478) mutation‐encoding site.^[^ ^32^ ^]^ The patient exhibited pronounced thrombocytopenia and anemia, closely resembling the phenotypes observed in the ABIN1 (Q478) mutant mice. Thus, our studies could provide an opportunity to develop strategies for the treatment of MDSs through IFN signaling modulation.

Apoptosis can be stimulated by two pathways: the P53‐involved intrinsic pathway and the Fas death receptor (FAS)‐induced extrinsic pathway (48). P53‐involved apoptosis is the main cause of MDSs induced by ribosomal protein S14 (Rps14) haploinsufficiency.^[^ ^8^, ^33^ ^]^ However, the role of FAS‐induced apoptosis in hematopoiesis has rarely been discussed. In this study, we observed that *Abin1^Q478H/Q478H^* MDFs were sensitive to TNFα/CHX (TC)‐induced extrinsic apoptosis (Figure S6a, Supporting Information). The levels of cleaved caspase 3 (a marker of apoptosis) were higher in *Abin1^Q478H/Q478H^* MDFs than in WT MDFs following exposure to TC treatment (Figure S6b, Supporting Information). Furthermore, we observed abnormal erythroid cell apoptosis in the *Abin1^Q478H/Q478H^* mice, suggesting that excessive extrinsic apoptosis may contribute to hematological defects in these mice (Figure S6c, Supporting Information). Although blocking extrinsic apoptosis by means of *Fadd* (encoding Fas associated via death domain) and *Casp8* (encoding caspase 8) gene knockout is possible, it should be noted that *Fadd^–/–^* and *Casp8^–/–^* mice still die during the perinatal period. Additionally, the hematopoietic deficiencies that appear in *Fadd^–/–^* and *Casp8^–/–^* mice cannot be ameliorated by blocking necroptosis.^[^ ^34^ ^]^ Therefore, completely blocking extrinsic apoptosis is unlikely to alleviate the hematopoietic deficiencies observed in *Abin1^Q478H/Q478H^* mice. However, we generated *Abin1^Q478H/Q478H^Ripk3^–/–^Fadd^+/–^* mice to investigate whether FADD expression levels contribute to MDS‐like disease development in *Abin1^Q478H/Q478H^* mice. Our results clearly demonstrated that FADD expression levels did not affect hematopoietic deficiencies in *Abin1^Q478H/Q478H^Ripk3^–/–^* mice (Figure S6d, Supporting Information). Thus, extrinsic pathway‐induced apoptosis is distinct from the P53‐involved intrinsic pathway in hematopoiesis.

MLKL and RIPK3 are key proteins involved in necroptosis, a cell death process that can be blocked through knockout of the *Mlkl* or *Ripk3* genes. However, MLKL and RIPK3 also have distinct functions in hematopoiesis. *Casp8^–/–^Ripk3^–/–^* double‐knockout mice exhibit higher levels of RBCs and platelets than *Casp8^–/–^Mlkl^–/–^* double‐knockout mice.^[^ ^34a^ ^]^ Our study revealed that RIPK3 regulates *Abin1^Q478H/Q478H^* anemia through necroptosis‐independent mechanisms. Previous studies have suggested that RIPK3 can disrupt the functions of HSCs and induce p38/p16‐mediated cellular senescence, which rely on reactive oxygen species production but not on MLKL.^[^ ^35^ ^]^ These findings demonstrate that the role of RIPK3 in hematopoiesis extends beyond its involvement in the necroptotic cell death pathway via the RIPK1–RIPK3–MLKL axis.

In terms of the relationship between RIPK3 and IFN‐I, previous research has shown that chronic activation of the IFN‐I pathway has detrimental effects on HSC self‐renewal and differentiation.^[^ ^36^ ^]^ Additionally, studies using *Irf2^−/^* ^−^ mice by the laboratory of Taku Sato have demonstrated the ability of type I IFNs to induce proliferation and depletion of HSCs.^[^ ^7b^ ^]^ In contrast, RIPK3 knockout has been reported to effectively prevent anemia in *Gpx4*‐deficient mice and counteract compensatory anemia in peripheral organs.^[^ ^23a^ ^]^ Furthermore, the absence of RIPK3 protein has been shown to prevent bone marrow failure in *Tak1^mut^Tnfr^−/−^* mice.^[^ ^23b^ ^]^ Although both IFN‐I and RIPK3 have been reported to regulate the hematopoietic system, the focus of IFN‐I studies is mainly on its regulation of HSCs, while RIPK3 emphasizes anemia. Furthermore, our experimental results demonstrate that the knockout of IFNAR1 can both rescue anemia and alleviate thrombocytopenia symptoms. However, RIPK3 only plays a necroptosis‐independent role in anemia, and it does not regulate thrombocytopenia. Therefore, these two proteins have different cellular targets, and their signaling pathway may also differ in different cell types, which requires further research to elucidate the underlying mechanisms.

Given that myelodysplastic syndrome (MDS) is an age‐related disease, the relationship between different types of MDS and age may vary.^[^ ^37^ ^]^ First, we conducted hematopoiesis‐related experimental analysis using *Abin^Q478H/Q478H^* mice of different ages. We observed that *Abin^Q478H/Q478H^* mice displayed a more pronounced anemia and thrombocytopenia at 10 months of age as compared with 5 months of age (Figure 1a). Although we did not have 18‐month‐old mice, we conducted an analysis on a 14‐month‐old *Abin^Q478H/Q478H^* mouse. Our analysis revealed that the concentration of red blood cells (5.2^x^10^12^/L) and platelets (3.86^x^10^11^/L) in its peripheral blood indicated severe hematopoietic defects. In the analysis of bone marrow cells, we observed a significant decrease in the total number of bone marrow cells in 10‐month‐old *Abin^Q478H/Q478H^* mice compared with 5‐month‐old *Abin^Q478H/Q478H^* mice (Figure S7a, Supporting Information). Flow cytometry analysis further revealed a notable decline in the population of LSK in *Abin^Q478H/Q478H^* mice, primarily attributed to a significant reduction in the numbers of HPC‐1 and HPC‐2 cells (Figure S7a, Supporting Information). These results suggest that the hematopoietic defect in *Abin^Q478H/Q478H^* mice worsens with increasing age over time.

Cellular senescence, considered to be one of the hallmarks of aging, is defined as a stable growth arrest predominantly mediated by cell cycle regulators p53, p21, and p16.^[^ ^38^ ^]^ Therefore, we hypothesized that the ABIN1(Q478H) mutation leads to cellular senescence and subsequently triggers an MDS‐like disease. We compared the levels of p16 and p53 proteins in HPC‐1, HPC‐2, MPP, and LT‐HSC cells of *Abin^Q478H/Q478H^* mice and WT mice. The results showed no significant differences in protein levels between them (Figure S7b, Supporting Information). Additionally, immunohistochemistry staining of bone marrow sections also showed no significant differences in the expression of p53 and p21 proteins in bone marrow cells of *Abin^Q478H/Q478H^* mice and WT mice (Figure S7c, Supporting Information). Furthermore, the analysis of RNA‐seq results and enrichment analysis of the cellular senescence‐related gene set provided additional evidence. The TPM expression data revealed that there were no significant fold differences in the expression levels of p16 (*Cdkn2a*), p21 (*Cdkn1a*), p53 (*Trp53*), and CD41 (*Itga2b*) proteins between HPC‐1, HPC‐2, MPP, and LT‐HSC cells of *Abin^Q478H/Q478H^* mice and WT mice. (Figure S7d–g, Supporting Information). This indicates that there is no upregulation of these senescence‐related markers in the ABIN(Q478H) mutant HSPC. Moreover, the heatmap generated using the TPM expression data of the cellular senescence‐related gene (GO:00 90398) set also showed no significant differences between *Abin^Q478H/Q478H^* and WT cells (Figure S7h, Supporting Information). Collectively, these experimental results suggest that there is no evidence of higher levels of cellular senescence in HPC‐1, HPC‐2, MPP, and LT‐HSC cells of *Abin^Q478H/Q478H^* mice compared with WT mice. Additionally, *Abin1* knockout mice and *Abin^Q478H/Q478H^* mice both showed anemia symptoms at 6 to 8 weeks of age, indicating that the ABIN1(Q478H) mutation does not lead to hematopoietic defects through promoting cellular senescence of HSPC cells. Taken together, these findings indicate that although the ABIN1(Q478H) mutation does not induce HSPC senescence as a mechanism for hematopoietic defects, the severity of hematopoietic defects in *Abin^Q478H/Q478H^* mice becomes more pronounced with increasing age.

In conclusion, we have identified the critical role of ABIN1 (Q478) in regulating hematopoiesis through its prevention of excessive type I IFN expression. Dysregulation of this process can lead to MDS‐like diseases. Thus, the blockade of type I IFN signaling may have therapeutic potential in patients with hematopoietic disorders. Furthermore, *Abin1^Q478H/Q478H^* mice represent a valuable model for studying MDSs and related hematopoietic disorders and can aid in the development of new treatments for hematopoietic defects. Overall, our findings provide new insights into the pathogenesis of hematopoietic diseases and offer potential avenues for future research and therapeutic interventions.

## 4 Experimental Section

### Mice

The mice used in this investigation were C57BL/6 genetic background and were maintained in a pathogen‐free (SPF) facility at the Shanghai Institute of Nutrition and Health, Chinese Academy of Sciences. Both male and female mice were used in this study. *Ripk1^K45A/K45A^*, *Ripk3^−/−^*, *Mlkl^−/−^*, *Ifnar1^−/−^* mouse lines have been described previously.^[^ ^11^, ^39^ ^]^ *Abin1^Q478H/Q478H^* mice were generated in exon 14 of the ABIN1 locus by the CRISPR‐Cas9 mutation system (Bioray Laboratories Inc., Shanghai, China). *Abin1^Q478H/Q478H^* mice genotyping primers: 5′‐GGGTCTGACTTGTAGGGTCG‐3′ and 5′‐CAGGCATACATGCAGGCAGA‐3′ amplified 324 bp DNA fragments for sequencing. Animal experiments were carried out following the guidelines of the Institutional Animal Care and Use Committee of Shanghai Institute of Nutrition and Health, Chinese Academy of Sciences (SYXK 2019‐0001).

### Flow Cytometry and Peripheral Blood Analysis

Single‐cell suspensions from the spleen and bone marrow were counted using counting slides (SD‐100, Nexcelom) in a Cellometer Mini Automated Cell Counter (Nexcelom). Surface antigens were stained with demonstrated conjugated primary antibodies in the staining buffer (1× PBS, 3% BSA, 1 mM EDTA, 0.1% NaN3) at 4 °C for 30 min. Antibodies used were as follows: APC TER‐119 (557 909, BD), PE CD71 (553 267,BD), Biotin CD3 (100 244, Biolegend), Biotin CD45R (13‐0452‐81, eBioscience),Biotin CD4 (13‐0041‐81, eBioscience), Biotin CD5 (13‐0051‐81, eBioscience), Biotin CD8a Biotin (13‐0081‐81, eBioscience),Biotin CD19 (13‐0193‐81, eBioscience),Biotin CD11b (13‐0112‐81, eBioscience),Biotin Ly‐6G (13‐5931‐81, eBioscience), Biotin TER‐119 (13‐5921‐81, eBioscience), PE‐Cy7 Streptavidin (25‐4317‐82, eBioscience), APC Ly‐6A/E (Sca‐1) (17‐5981‐82, eBioscience), APC‐EFLUOR CD117 (C‐KIT) (47‐1172‐82, eBioscience). After staining, cells were washed once with 1× PBS and quickly analyzed by a cytoflex S flow cytometer (cytoflex S, Beckman Coulter). All analyses were performed using CytExpert software (CytExpert, Beckman Coulter, Inc.).

An auto hematology analyzer (BC‐2800Vet, Mindray) was used to analyze diluted peripheral blood that had been collected in an anticoagulation tube and diluted in EDTA buffer (0.5 M EDTA, pH 8.0) at a quantitative ratio of 1:1.

### Cell Culture

All primary MDFs were cultured from mice that were born within one day. Primary MDFs were cultured in high‐glucose Dulbecco's modified Eagle's medium (SH30243.LS, Hyclone) supplemented with 10% fetal bovine serum, 2 mM glutamine, 1% penicillin, and 100 µg ml^−1^ streptomycin. HEK293T cells were cultured in complete DMEM. All primary BM‐derived macrophages (BMDMs) were cultured in 10‐cm dishes with DMEM containing 20% FBS and L929 conditional medium isolated from mouse tibia and femur.

Colony‐forming unit (CFU) assays of lineage‐negative‐Sca1^+^‐Kit^+^ (LSK) mouse bone marrow was cultured by MethoCult GF M3434 (STEMCELL) for 7 days after being selected by MoFlo Astrios EQ (Beckman).

To assay for stem cells using the CAFC assay, HSPC cells were cocultured on an MS‐5 monolayer in IMDM containing 12.5%FCS, 12.5% horse serum, 1 µM hydrocortisone, 4 mM L‐glutamine, and 100 U ml^−1^ penicillin/streptomycin. The cultures were demidepopulated each week. After 3–4 weeks in culture, total cobblestone areas were counted.

### Western Blot and Immunoprecipitation

After stimulation, cells were reaped and washed with PBS, then lysed with RIPA lysis buffer (50 mM Tris‐HCl (pH 7.4), 150 mM NaCl, 2 mM EDTA, 1% NP‐40, 0.1% SDS, Protease inhibitor Cocktail (4 693 132 001, Roche), Phosphatase inhibitor Cocktail 3 (P0044‐1ML, Sigma)) for 30 min on ice. The lysates were centrifuged for 20 min at 13,200 g, 4 °C, then quantified by BCA kit (P0010S, Beyotime), and mixed with SDS sample buffer (250 mM Tris‐Cl (pH 6.8), 10% SDS, 30% Glycerol, 5% β‐mercaptoethanol, 0.02% Bromophenol blue) followed by boiling at 100 °C for 10 min. The proteins were separated by SDS‐PAGE, and then transferred to PVDF membrane (IPVH00010, Millipore) at 110 V for 2 h.

With 5% skimmed milk in PBST 0.1%, membranes were blocked for one hour, then membranes were washed three times with PBST 0.1% for 5 min, and incubated in PBST 0.1% containing primary antibodies at 4 °C overnight. The proteins were detected by chemiluminescent substrate (34 080, Thermo Scientific) using Tanon 5200 Multi Luminescent Imaging Workstation (Tanon).

For TNF‐RSC immunoprecipitation, immortalized MDFs were stimulated with flag‐TNF‐α (100 ng ml^−1^) for the PBS and subsequently lysed in NP‐40 buffer at 4 °C for 1 h with rotation. The lysates were cleared by centrifugation and then incubated with FLAG‐tagged beads (A2220, Sigma) for 16 h. At least three times, the beads were washed with ice‐cold NP‐40 buffer. Proteins were eluted with 40 µL 2x SDS loading sample buffer.

For coimmunoprecipitation assays, 293T were transfected with the indicated plasmids using Lipofectamine 2000 for 24 hours. Then collect 293T lysed with 1% NP‐40 buffer ((50 mM Tris‐HCl (pH8.0), 150 mM NaCl, 2 mM EDTA, 1% NP‐40 containing protease inhibitors) for 30–45 min on ice. The remaining cell extracts were cleared by centrifugation and then incubated with FLAG‐tagged beads (A2220, Sigma) for 16 h after some were saved for input analysis. The beads were washed with ice‐cold NP‐40 buffer at least three times. Proteins were eluted with 40 µL 2x SDS loading sample buffer.

The primary antibodies used for western blot: ABIN1 (15104‐1‐AP, Proteintech), anti‐RIPK1 (610 459, BD Biosciences), anti‐phosphorylated RIPK1 (31122S, Cell Signaling Technology), anti‐RIPK3 (2283, Prosci), anti‐phosphorylated RIPK3 (ab195117, Abcam), anti‐MLKL (AP14272b, Abgent), anti‐phosphorylated MLKL (ab196436, Abcam), anti‐β‐actin (3779, Prosci), anti‐GAPDH (G9545, Sigma), IRF3 (1:1000, Cell Signaling Technology, 4302S), and p‐IRF3 (1:1000, Cell Signaling Technology, 4947S). The Smac mimetic and poly (I:C) were purchased from Sigma. TNF‐α (410‐MT‐050) was procured from R&D. Nec‐1 and zVAD were purchased from MCE. Flag‐beads (A2220) were purchased from Sigma. Flag‐TNF‐α (ALX‐522‐009‐C050) was purchased from ENZO.

### Cell Survival Assay

CellTiter‐Glo Luminescent Cell Viability Assay Kit (G7572, Promega) was used to measure cell survival, and a microplate luminometer was used to measure luminescence (Thermo Scientific).

### RNA‐Seq and Quantitative RT‐PCR

Total RNAs were extracted using Trizol (9108, Takara) from both sorted lineage‐negative bone marrow cells and amplified HPC‐1, HPC‐2, MPP, and LT‐HSC of 5‐month‐old WT and *Abin^Q478H/Q478H^* mice. RNA samples were quantified using a Qubit 3.0 Fluorometer (Life Technologies, CA, USA), and RNA integrity was assessed using a 4200 TapeStation (Agilent Technologies, CA, USA). The RNA sequencing library was constructed and sequenced using the Illumina Novaseq PE150 platform. Gene set enrichment analysis was performed on GSEA_4.3.2, and visualization of differentially expressed genes was implemented by the pheatmap R package and ggplot R package. The RNA‐seq raw data was available at GEO (GSE220301) and GEO (GSE245029).

As directed by the manufacturer, total RNA was extracted using Trizol reagent (9108, Takara). After quantification, 1 µg total RNA was reverse transcribed to complementary DNA (RR047A, Takara). Transcript levels of indicated genes were quantified by qPCR on a QuantStudio 6 PCR instrument with SYBR Green (RR420A, Takara). The sequences of primers were shown as follows.

GAPDH‐F:TGTGTCCGTCGTGGATCTGA;
GAPDH‐R:GGTCCTCAGTGTAGCCCAAGL;
IFNγ‐F:ACAGCAAGGCGAAAAAGGAT;
IFNγ‐ R:TGAGCTCATTGAATGCTTGG;
IFNα‐F:CCTTCCACAGGATCACTGTGTACCT;
IFNα‐R:TTCTGCTCTGACCACCTCCC;
IFNβ‐F:CACAGCCCTCTCCATCAACT;
IFNβ‐R:TCCCACGTCAATCTTTCCTC;
IFNAR1‐F:AGCCACGGAGAGTCAATGG;
IFNAR1‐R:GCTCTGACACGAAACTGTGTTTT;
IFNAR2‐F:CTTCGTGTTTGGTAGTGATGGT;
IFNAR2‐R:GGGGATGATTTCCAGCCGA;
PTPN11‐F:AGAGGGAAGAGCAAATGTGTCA;
PTPN11‐R:CTGTGTTTCCTTGTCCGACCT;
ADAR‐F:TACCTGAACACCAACCCCGTA;
ADAR‐R:GAGACTGGCCCCTGTTACTG;
IRF7‐F:GAGACTGGCTATTGGGGGAG;
IRF7‐R:GACCGAAATGCTTCCAGGG;
STAT2‐F:TCCTGCCAATGGACGTTCG;
STAT2‐R:GTCCCACTGGTTCAGTTGGT.

### BM Transplantation Assay

Using the CIX3 (Xstrahl) irradiator, all C57BL/6 recipient mice received 9 Gy of total body radiation. Irradiated recipients were reconstituted by intravenous injection of 2.5×10^6^ bone marrow cells from the femurs and tibias of the indicated genotype mice, which were two months old. After reconstitution, recipients were sacrificed after 100 days.

### Histopathology

The mice livers and spleens were fixed in 4% paraformaldehyde for hematoxylin and eosin staining. The mice femurs and tibias were decalcified using 10% EDTA decalcifying fluid and then subjected to hematoxylin and eosin staining and reticular fibers staining.

### Statistical Analysis

GraphPad Prism 8.0 was used to analyze the data using the two‐tailed unpaired Student t‐test. Please refer to the figure legends for the description of statistical significance. Differences were considered statistically significant when the P value was less than 0.05. The following symbols were used to indicate levels of significance: **** for P < 0.0001, *** for P < 0.001, ** for P < 0.01, * for P < 0.05, and ns for not significant.

### Ethics Approval

All animal experiments were conducted in accordance with the guidelines of the Institutional Animal Care and Use Committee of Shanghai Institute of Nutrition and Health, Chinese Academy of Sciences.

## Conflict of Interest

The authors declare no conflict of interest.

## Author Contributions

X.H.W. and H.B.Z. designed the study; X.H.W performed most of experiments and data analyses. Y.W. conducted analyses of the functionality of hematopoietic stem cells. B.Y.C analyzed clinical data from a publicly available dataset. Y.B.L., L.F. and H.W.Z. conducted the biochemical analysis of cell death. Y.J.O., X.X.W., X.M.L., L.X.W., W.W.R., J.L.L., X.M.Z., L.L.G., and H.L. assisted with mouse breeding and analysis. L.W., and A.K.L. provided experimental materials and contributed to critical discussion. Z.C.W. and M.L provided intellectual input and coordinated the project; X.H.W, M.L and H.B.Z wrote the paper with assistance from O.Y.J; H.B.Z supervised the project.

## Supporting information

Supporting Information

## Data Availability

The data that support the findings of this study are openly available in GEO at https://www.ncbi.nlm.nih.gov/geo/query/acc.cgi?acc=GSE220301, reference number 220301; https://www.ncbi.nlm.nih.gov/geo/query/acc.cgi?acc=GSE245029,reference number 245029.
