# Supplementary material for: ABIN1 (Q478) is Required to Prevent Hematopoietic Deficiencies through Regulating Type I IFNs Expression
Source: Adv Sci (Weinh). 2023 Nov 27;11(3):2303555. doi: 10.1002/advs.202303555 (PMC10797436; doi:10.1002/advs.202303555)
Supplement: Supplementary file 1 — Supporting Information [file ADVS-11-2303555-s001.pdf]

ADVANCED SCIENCE
Open Access

Supporting Information

for *Adv. Sci.*, DOI 10.1002/advs.202303555

ABIN1 (Q478) is Required to Prevent Hematopoietic Deficiencies through Regulating Type I IFNs Expression

Xuanhui Wu, Yong Wang, Bingyi Chen, Yongbo Liu, Fang Li, Yangjing Ou, Haiwei Zhang, Xiaoxia Wu, Xiaoming Li, Lingxia Wang, Wuwei Rong, Jianling Liu, Mingyan Xing, Xiaoming Zhao, Han Liu, Lingling Ge, Ankang Lv, Lan Wang, Zhichao Wang, Ming Li* and Haibing Zhang**

Supporting Information:

Supplemental Figure Legends

Supplemental Figure S1. Hematopoietic defects in *Abin1*$^{Q478H/Q478H}$ mice

(**a**) Differential expression of TNIP1 in $CD34^+$ cells from healthy individuals and from patients with del(5q) myelodysplastic syndrome (MDS), from GEO(GSE19429) expression microarrays. (n = 17–46 patients/group)

(**b**) Overall survival of patients with MDS stratified by CD34 expression. (n = 80 patients; TNIP1-Low: Z score < –0.65; TNIP1-Normal: –0.65 < Z score <0.65; TNIP1-High: Z score > 0.65)

(**c**) Schematic diagram of the *Abin1* gene and ABIN1 (Q478H).

(**d**) Immunoblot analysis of ABIN1 in whole-cell lysates of spleen and bone marrow (BM).

(**e**) Body weight of mice at the indicated age. (n≥ 6 mice/group)

(**f**) Sizes of the lymph nodes and spleens of 5-month-old wild-type (WT) and *Abin1*$^{Q478H/Q478H}$ mice.

(**g**) Analysis of peripheral blood white blood cells (WBCs) from WT and *Abin1*$^{Q478H/Q478H}$ mice. (n ≥ 5 mice /group).

(**h–i**) Lymph, Mon, and Gran at 5 months (**h**) and 10 months of age (**i**). (n ≥ 9 mice /group).

(**j**) Quantification of the frequencies of $CD3^+$ and $B220^+$ cells in 10-month-old WT and *Abin1*$^{Q478H/Q478H}$ mouse spleens. (n ≥ 4 mice /group)

(**k**) Quantification of splenic cell frequency in 5-month-old WT and *Abin1*$^{Q478H/Q478H}$ mice at the indicated erythroid differentiation stages (RI, proerythroblasts; RII, basophilic erythroblasts; RII, chromatophilic erythroblasts; RIV, orthochromatophilic erythroblasts). (n ≥ 4 mice /group)

(**l**) Enlarged hematoxylin and eosin-stained image of liver tissue from a 10-month-old *Abin1*$^{Q478H/Q478H}$ mouse.

(**m**) Hematoxylin and eosin staining of the liver tissue. Representative images of at least three mice of the indicated age per group. The panel data were analyzed using the two-tailed unpaired Student t-test, and statistical significance was indicated as follows: **** for P < 0.0001, *** for P < 0.001, ** for P < 0.01, and * for P < 0.05.

Figure S1

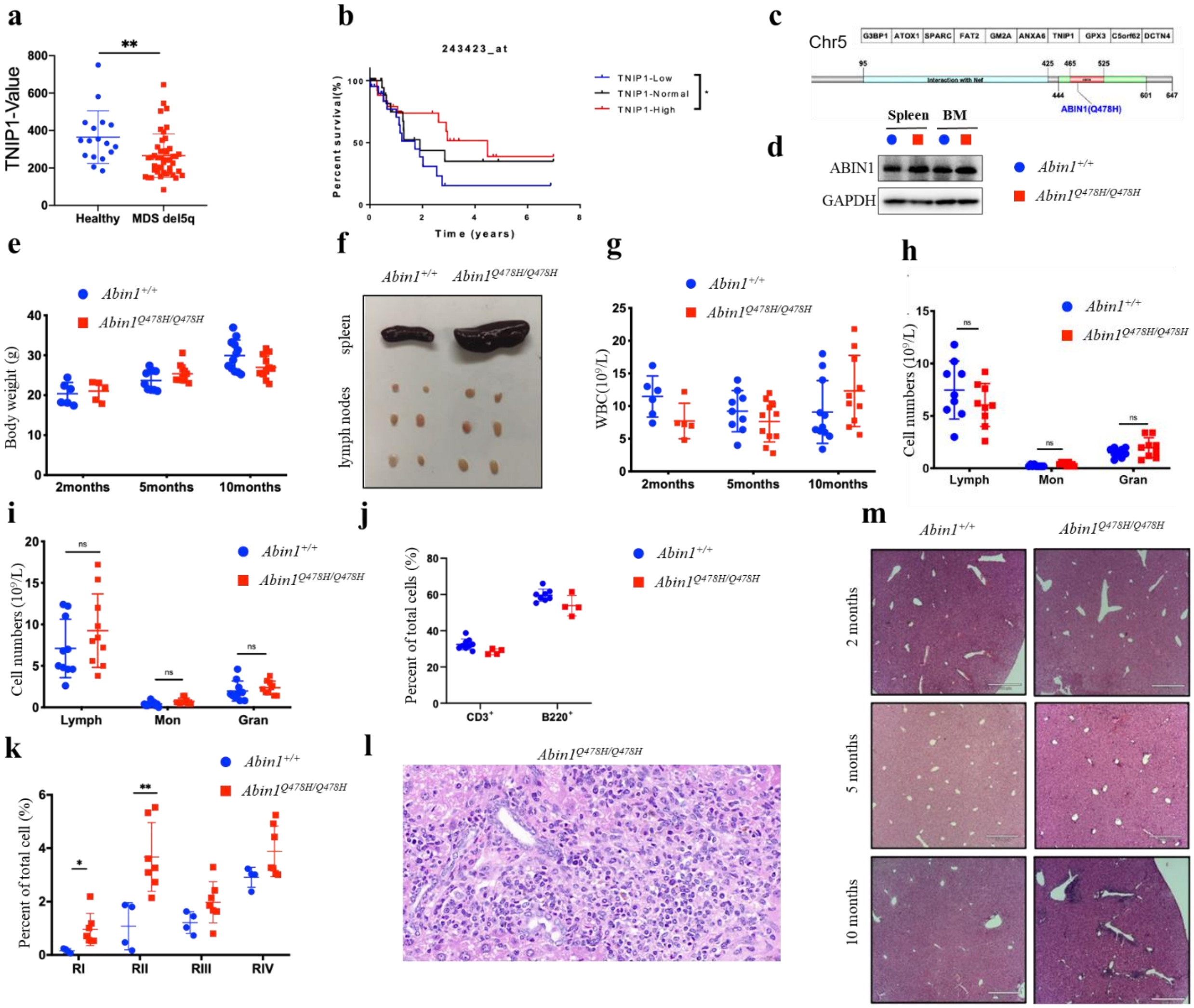

Supplemental Figure S2. ***Abin1$^{Q478H/Q478H}$*** **mice had deficient bone marrow development**

(**a**) Reticular fiber-stained images of bone marrow (BM) from 5-month-old wild-type (WT) and *Abin1$^{Q478H/Q478H}$* mice.

(**b**) Quantification of the frequencies of megakaryocyte-erythrocyte progenitors (MEPs), granulocyte-macrophage progenitors (GMPs), and common myeloid progenitors (CMPs) in BM cells from 5-month-old WT and *Abin1$^{Q478H/Q478H}$* mice. (n ≥ 7 mice/group).

(**c**) The representative flow cytometry profiles of the LSK and LSK-subpopulation cells in the bone marrow of WT and *Abin1$^{Q478H/Q478H}$* mice at 5 months old were obtained.

(**d**) The frequencies of the LSK and LSK-subpopulation cells in the bone marrow of WT and *Abin1*$^{Q478H/Q478H}$ mice at 5 months old and 9 months old were quantified (n ≥ 3 mice/group).
(**e**) Quantification of the total colony number and total cell number in colony-forming unit (CFU) assays. LSK-subpopulation cells (3×10^3) isolated from 5-month-old WT and *Abin1*$^{Q478H/Q478H}$ BM cells were plated for each assay. (n = 3 mice/group)
(**f**) Cobblestone-area forming cell (CAFC) assays of LSK-subpopulation cells isolated from 5-month-old WT and *Abin1*$^{Q478H/Q478H}$ BM cells were plated for each assay. (n = 3 mice/group)
(**g**) photo of cobblestone-area forming cell (CAFC) assays
(**h**) Quantification of the frequencies of spleen cells at the indicated erythroid differentiation stages in 2-month-old WT and *Abin1*$^{Q478H/Q478H}$ BM cell-recipient mice. (n ≥ 4 mice/group)
(**i**) Quantification of myeloid frequencies ($CD11b^+$ and $Gr\text{-}1^+$) in spleen cells of 2-month-old WT and *Abin1*$^{Q478H/Q478H}$ BM cell-recipient mice 100 days after cell transplantation. (n ≥ 4 mice/group) The panel data were analyzed using the two-tailed unpaired Student t-test, and statistical significance was indicated as follows: **** for P < 0.0001, *** for P < 0.001, ** for P < 0.01, and * for P < 0.05.

Figure S2

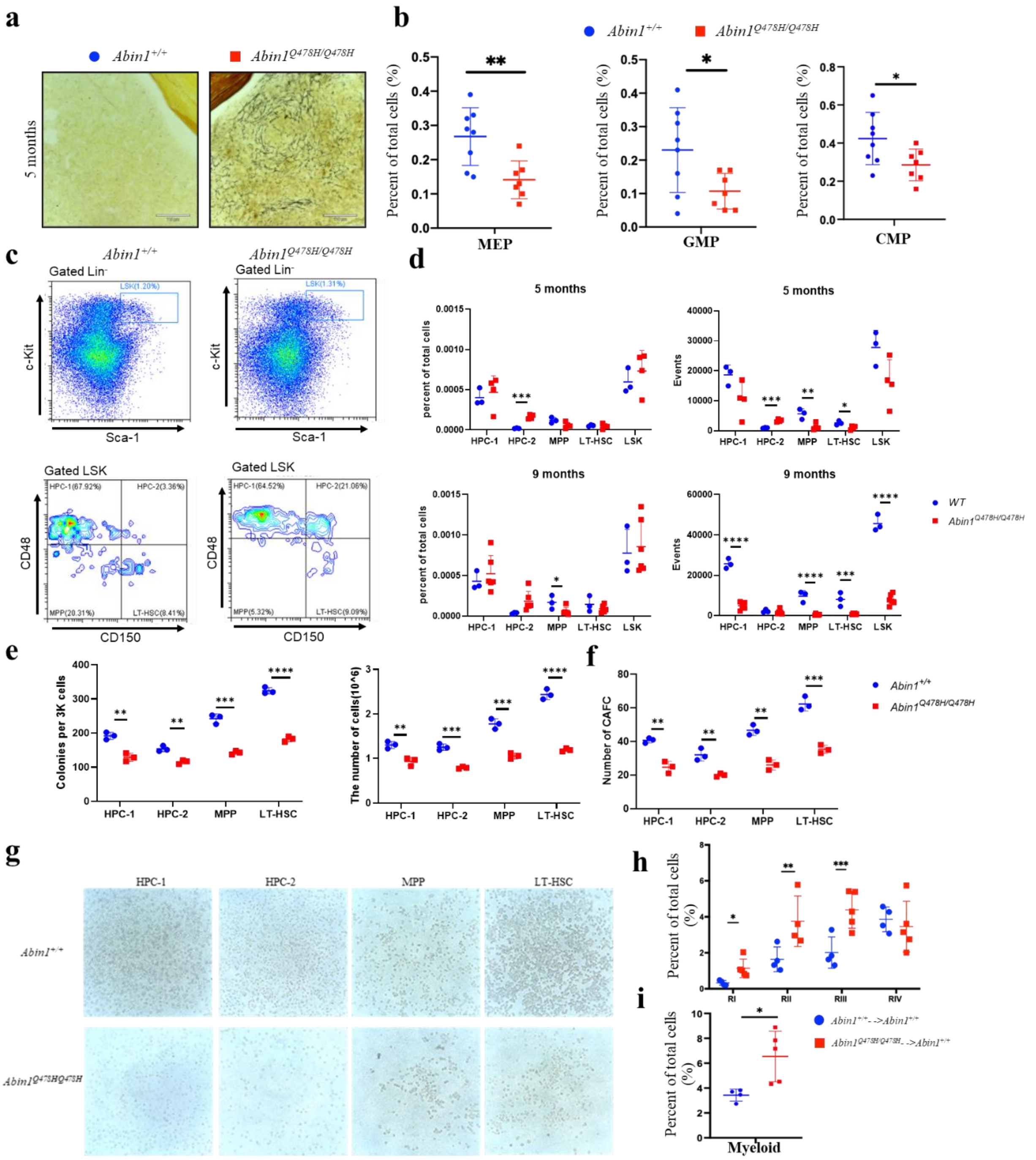

Supplemental Figure S3. Ripk3 and its necroptosis function are not the reason for *Abin1*$^{Q478H/Q478H}$ developed thrombocytopenia

(**a**) Cell survival of WT and *Abin1*$^{Q478H/Q478H}$ primary BMDMs treated with TNFα/Smac/zVAD (TSZ) or LPS/zVAD (LZ) in the presence or absence of Nec-1s

(**b**) Quantification of the frequency of splenic cells in 10-month-old *Abin1*$^{Q478H/Q478H}$ and *Abin1*$^{Q478H/Q478H}$*Ripk3*$^{-/-}$ mice at the indicated erythroid differentiation stages (RI, proerythroblasts; RII, basophilic erythroblasts; RII, chromatophilic erythroblasts; RIV, orthochromatophilic erythroblasts). (n ≥ 4 mice/group)

(**c**) Femurs and tibias of 10-month-old *Abin1*$^{Q478H/Q478H}$ and *Abin1*$^{Q478H/Q478H}$*Ripk3*$^{-/-}$ mice.

(**d**) Spleen weights of 10-month-old *Abin1*$^{Q478H/Q478H}$, *Abin1*$^{Q478H/Q478H}$*Ripk3*$^{-/-}$, *Abin1*$^{Q478H/Q478H}$*Ripk1*$^{K45A/K45A}$, and *Abin1*$^{Q478H/Q478H}$*Mlkl*$^{-/-}$ mice. (n ≥ 10 mice/group)

(**e–f**) Peripheral blood analysis, including platelet (PLT) count (**d**) and hemoglobin level (**e**), of 10-month-old *Abin1*$^{Q478H/Q478H}$, *Abin1*$^{Q478H/Q478H}$*Ripk3*$^{-/-}$, *Abin1*$^{Q478H/Q478H}$*Ripk1*$^{K45A/K45A}$, and *Abin1*$^{Q478H/Q478H}$*Mlkl*$^{-/-}$ mice. (n ≥ 12 mice/group)

The panel data were analyzed using the two-tailed unpaired Student t-test, and statistical significance was indicated as follows: **** for P < 0.0001, *** for P < 0.001, ** for P < 0.01, and * for P < 0.05.

Figure S3

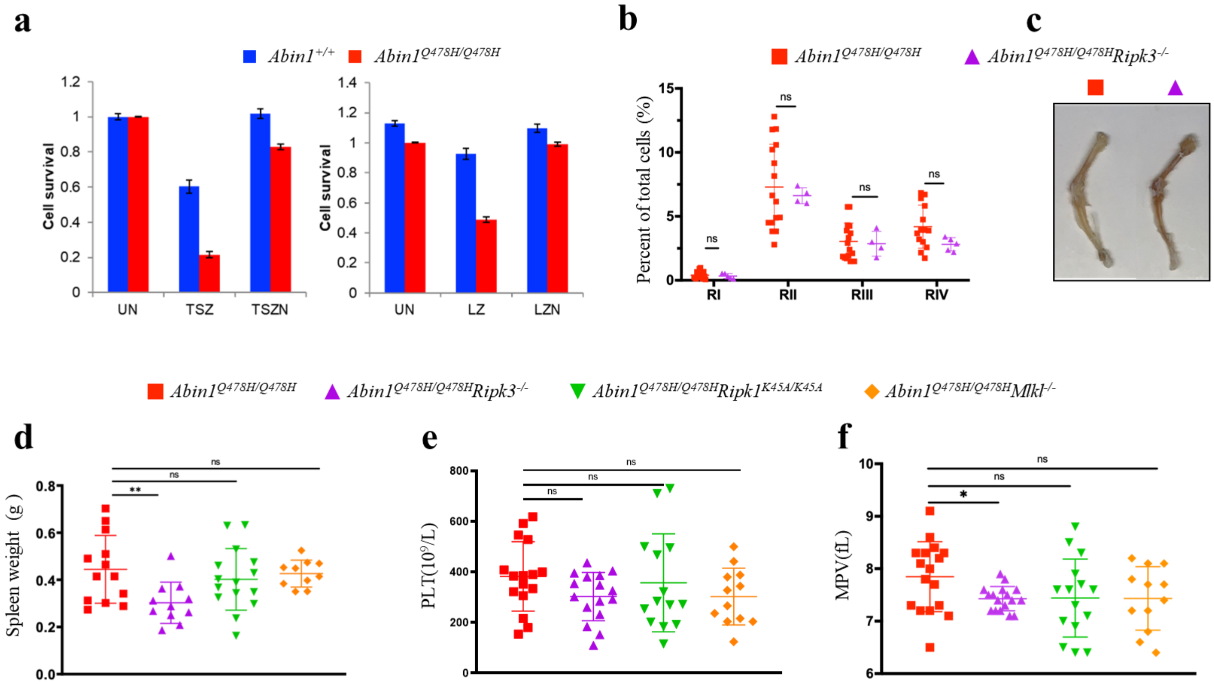

Supplemental Figure S4. Interferon signaling is crucial for the manifestation of hematopoietic defects in *Abin1*$^{Q478H/Q478H}$ mice

(**a**) Gene ontology related to interferon enrichment analysis of amplified HPC-1, HPC-2, MPP, and LT-HSC cells

(**b**) Peripheral blood analysis of 9-month-old *Abin1*$^{Q478H/Q478H}$ and *Abin1*$^{Q478H/Q478H}$*Ifnar1*$^{-/-}$ mice, including red blood cell (RBC) count, hemoglobin (HGB) level, platelet (PLT) count, and mean platelet volume (MPV). (n ≥ 7 mice/group)

Figure S4

a

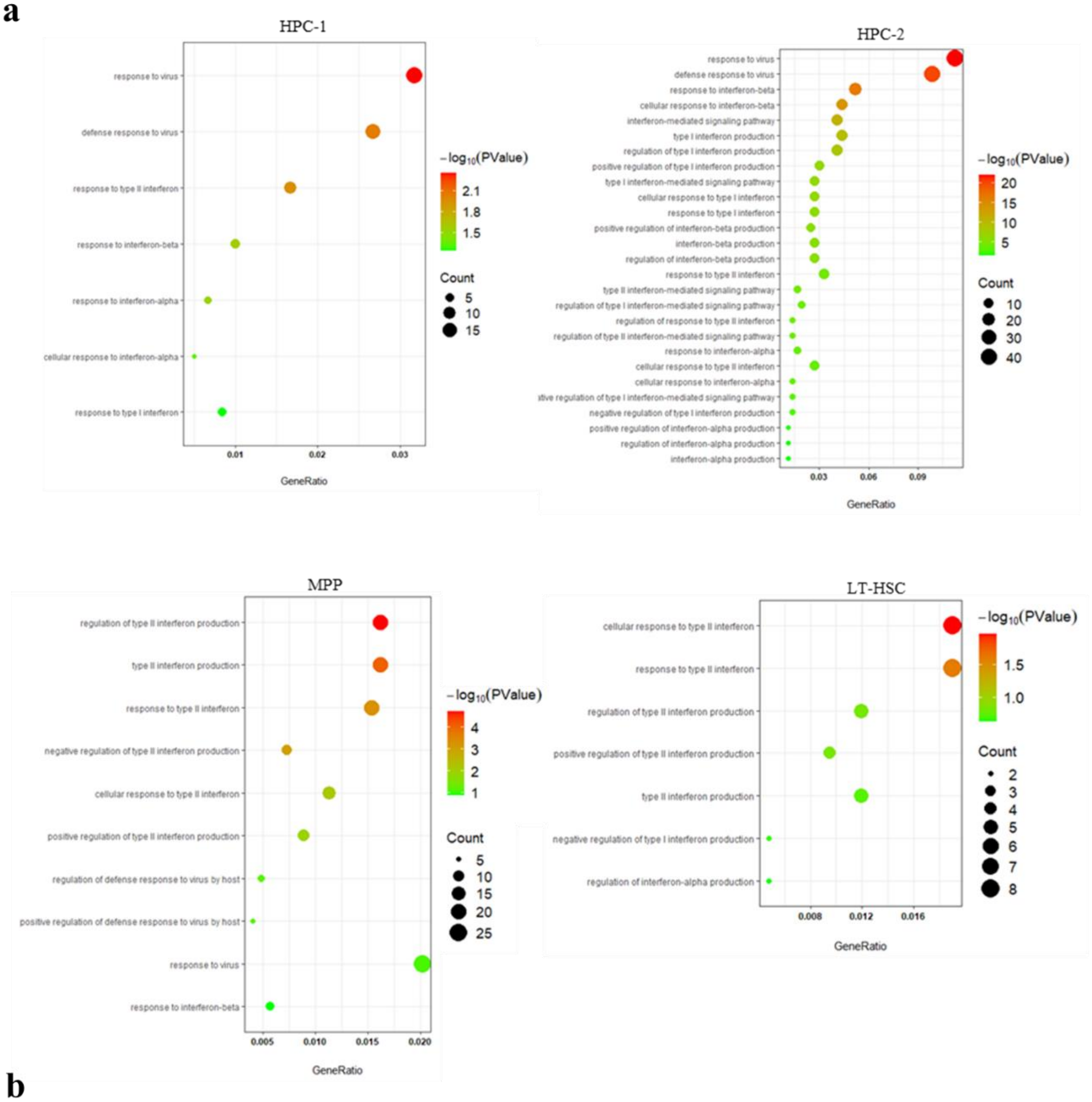
HPC-1
response to virus
defense response to virus
response to type II interferon
response to interferon-beta
response to interferon-alpha
cellular response to interferon-alpha
response to type I interferon
0.01
0.02
0.03
GeneRatio
-log10(PValue)
2.1
1.8
1.5
Count
5
10
15
HPC-2
response to virus
defense response to virus
response to interferon-beta
cellular response to interferon-beta
interferon-mediated signaling pathway
type I interferon production
regulation of type I interferon production
positive regulation of type I interferon production
type I interferon-mediated signaling pathway
cellular response to type I interferon
response to type I interferon
positive regulation of interferon-beta production
interferon-beta production
regulation of interferon-beta production
response to type II interferon
type II interferon-mediated signaling pathway
regulation of type I interferon-mediated signaling pathway
regulation of response to type II interferon
regulation of type II interferon-mediated signaling pathway
response to interferon-alpha
cellular response to type II interferon
cellular response to interferon-alpha
negative regulation of type I interferon production
positive regulation of interferon-alpha production
regulation of interferon-alpha production
interferon-alpha production
0.03
0.06
0.09
GeneRatio
-log10(PValue)
20
15
10
5
Count
10
20
30
40
MPP
regulation of type II interferon production
type II interferon production
response to type II interferon
negative regulation of type II interferon production
cellular response to type II interferon
positive regulation of type II interferon production
regulation of defense response to virus by host
positive regulation of defense response to virus by host
response to virus
response to interferon-beta
0.005
0.010
0.015
0.020
GeneRatio
-log10(PValue)
4
3
2
1
Count
5
10
15
20
25
LT-HSC
cellular response to type II interferon
response to type II interferon
regulation of type II interferon production
positive regulation of type II interferon production
type II interferon production
negative regulation of type I interferon production
regulation of interferon-alpha production
0.008
0.012
0.016
GeneRatio
-log10(PValue)
1.5
1.0
Count
2
3
4
5
6
7
8

b

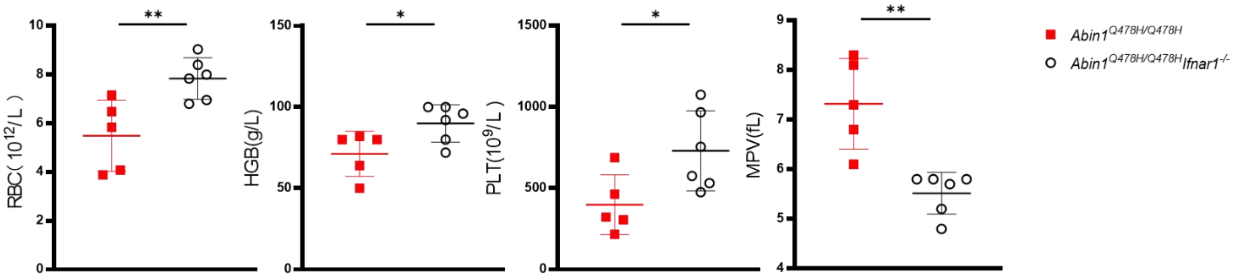
**
RBC(10^12/L)
10
8
6
4
2
0
*
HGB(g/L)
150
100
50
0
*
PLT(10^9/L)
1500
1000
500
0
**
MPV(fL)
9
8
7
6
5
4
Abin1^Q478H/Q478H
Abin1^Q478H/Q478H Ifnar1^-/-

Supplemental Figure S5. Schematic overview showing ABIN1 prevents hematopoietic deficiencies by regulating the expression of type I interferons.

Abin1$^{Q478H/Q478H}$ knock-in mice with a mutation that disrupts the polyubiquitin-binding site exhibit hematopoietic deficiency. Deficiency of *Ripk3* could alleviate anemia. However, co-deletion of *Ifnar1* greatly rescued anemia, thrombocytopenia, and splenomegaly in the *Abin1*$^{Q478H/Q478H}$ mice, indicating that ABIN1 maintains normal hematopoiesis by regulating type I interferon signaling and RIPK3-mediated necroptosis-independent function.

Figure S5

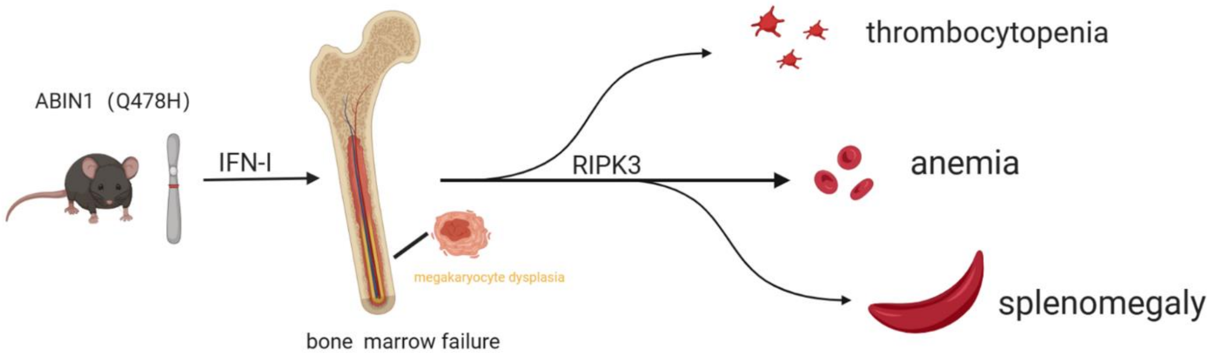

Supplemental Figure S6. Hematopoietic deficiencies in *Abin1*$^{Q478H/Q478H}$ *Ripk3*$^{-/-}$ mice are independent of FADD expression levels.

(**a**) Cell survival of wild-type (WT) and *Abin1*$^{Q478H/Q478H}$ primary mouse dermal fibroblasts (MDFs) treated with TNFα/cycloheximide (TC) for the indicated periods.

(**b**) Western blot analyses of wild-type (WT) and *Abin1*$^{Q478H/Q478H}$ primary mouse dermal fibroblasts (MDFs) treated with TNFα/cycloheximide (TC) for the indicated periods.

(**c**) With 100× magnification, Giemsa-stained bone marrow cytospins from 5-month-old *Abin1*$^{Q478H/Q478H}$ mice displayed abnormal apoptosis in immature red cell.

(**d**) Red blood cells (RBCs) of 5-month-old WT, *Abin1*$^{Q478H/Q478H}$, *Abin1*$^{Q478H/Q478H}$*Ripk3*$^{-/-}$, *Abin1*$^{Q478H/Q478H}$*Ripk3*$^{-/-}$*Fadd*$^{+/-}$ mice. (n ≥ 7 mice/group). The panel data were analyzed using the two-tailed unpaired Student t-test, and statistical significance was indicated as follows: **** for P < 0.0001, *** for P < 0.001, ** for P < 0.01, and * for P < 0.05.

Figure S6

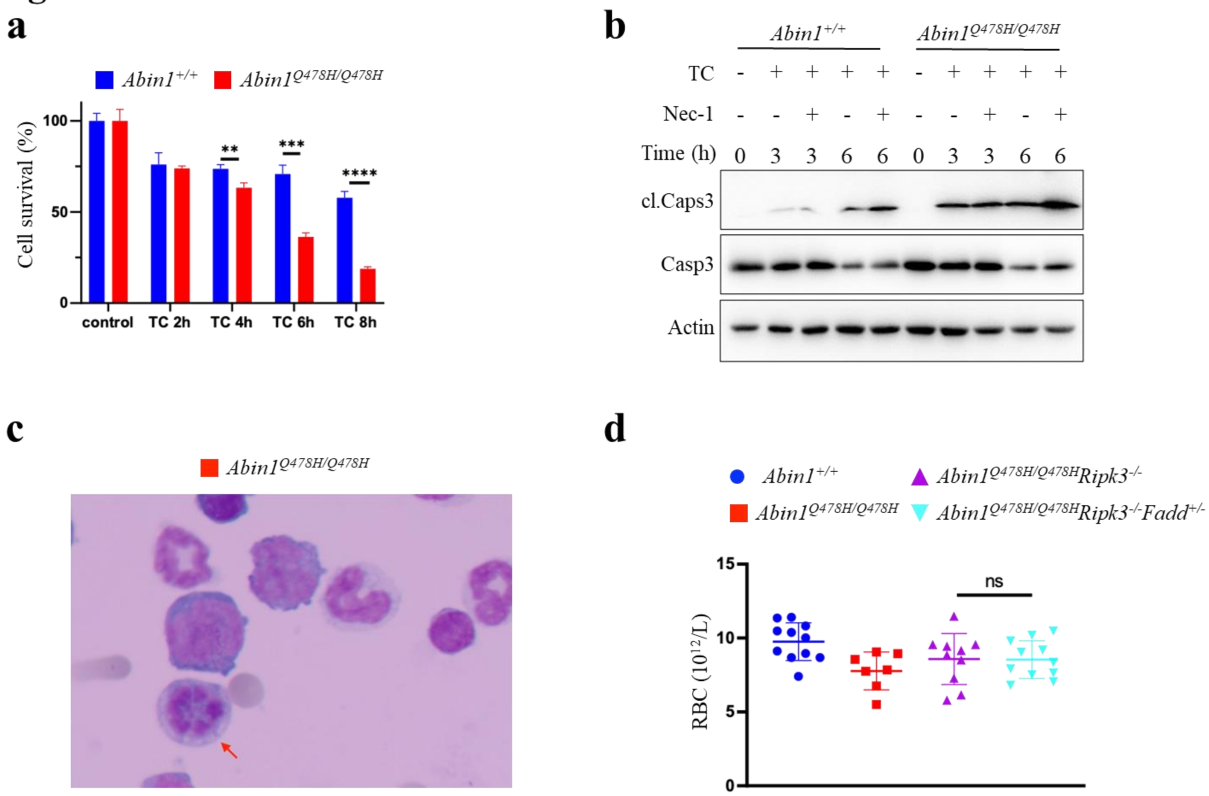

Supplemental Figure S7. The relationship between hematopoietic defects and cellular senescence in *Abin1*$^{Q478H/Q478H}$ mice

(**a**) The frequencies of the LSK and LSK-subpopulation cells in the bone marrow of WT and *Abin1*$^{Q478H/Q478H}$ mice at 5 months old and 9 months old were quantified (n ≥ 3 mice/group).

(**b**) Immunoblot analysis of P53 and P16 in whole-cell lysates of amplified HPC-1, HPC-2, MPP, and LT-HSC cells from 5-month-old WT and *Abin1*$^{Q478H/Q478H}$ mice.

(**c**) Immunofluorescence staining of P53 and P21 proteins in 10-month-old WT and *Abin1*$^{Q478H/Q478H}$ mice, with a magnification of 40×(n ≥ 3 mice/group).

(**d**) Expression levels of cdkn2a, cdkn1a, Trp53, and Itga2b in amplified HPC-1.

(**e**) Expression levels of cdkn2a, cdkn1a, Trp53, and Itga2b in amplified HPC-2.

(**f**) Expression levels of cdkn2a, cdkn1a, Trp53, and Itga2b in amplified MPP.

(**g**) Expression levels of cdkn2a, cdkn1a, Trp53, and Itga2b in amplified LT-HSC.

(**h**) Differentially expressed genes related to cell senescence were identified through expression profiling of Lin^{-} BM cells from 5-month-old WT and *Abin1*$^{Q478H/Q478H}$ mice

The panel data were analyzed using the two-tailed unpaired Student t-test, and statistical significance was indicated as follows: **** for P < 0.0001, *** for P < 0.001, ** for P < 0.01, and * for P < 0.05.

Figure S7

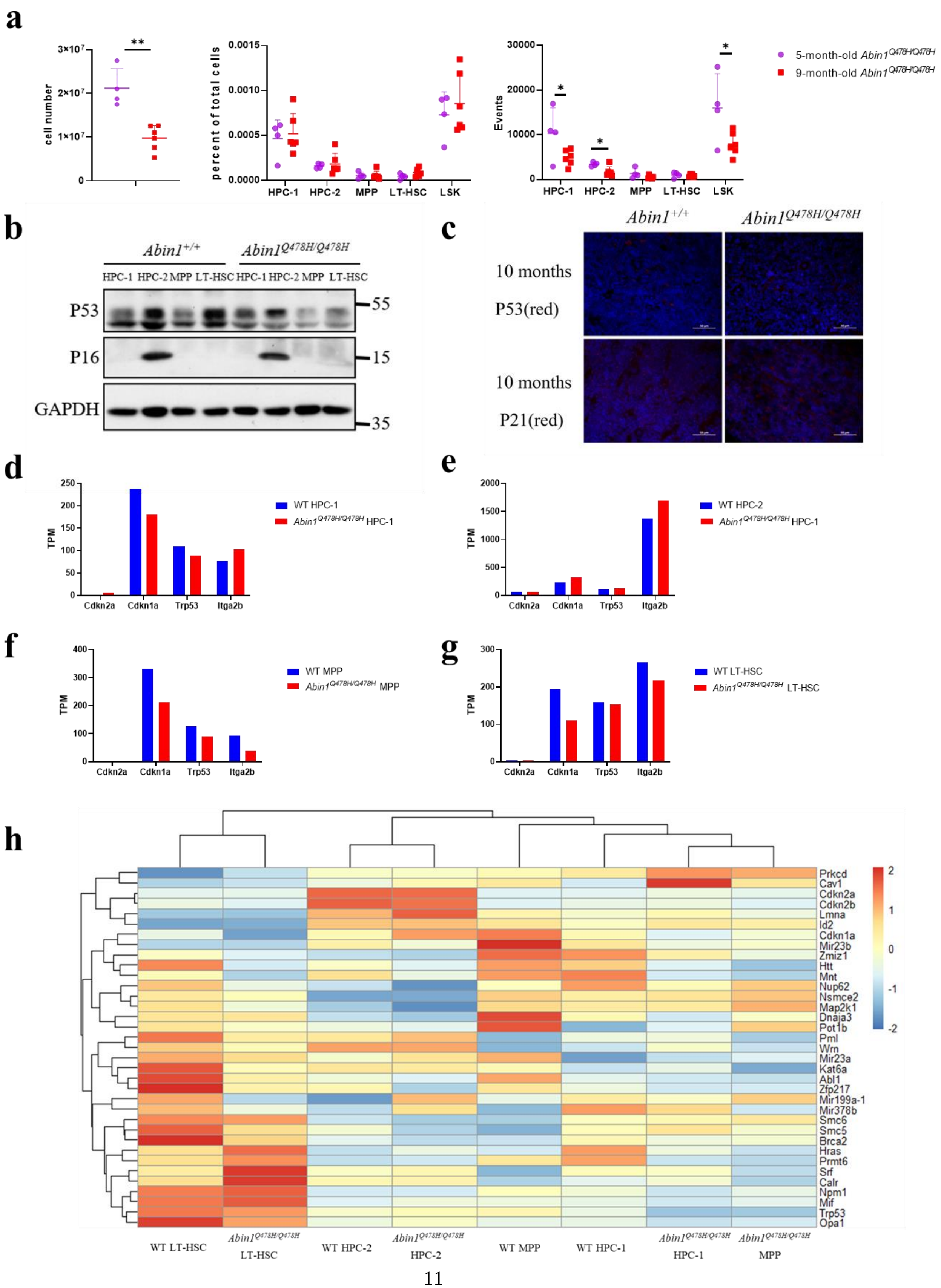
a
cell number
**
percent of total cells
Events
HPC-1 HPC-2 MPP LT-HSC LSK
5-month-old Abin1Q478H/Q478H
9-month-old Abin1Q478H/Q478H
b
Abin1+/+
Abin1Q478H/Q478H
HPC-1 HPC-2 MPP LT-HSC HPC-1 HPC-2 MPP LT-HSC
P53
P16
GAPDH
55
15
35
c
10 months
P53(red)
10 months
P21(red)
d
TPM
Cdkn2a Cdkn1a Trp53 Itga2b
WT HPC-1
Abin1Q478H/Q478H HPC-1
e
WT HPC-2
Abin1Q478H/Q478H HPC-1
f
WT MPP
Abin1Q478H/Q478H MPP
g
WT LT-HSC
Abin1Q478H/Q478H LT-HSC
h
Prkcd
Cav1
Cdkn2a
Cdkn2b
Lmna
Id2
Cdkn1a
Mir23b
Zmiz1
Htt
Mnt
Nup62
Nsmce2
Map2k1
Dnaja3
Pot1b
Pml
Wrn
Mir23a
Kat6a
Abl1
Zfp217
Mir199a-1
Mir378b
Smc6
Smc5
Brca2
Hras
Prmt6
Srf
Calr
Npm1
Mif
Trp53
Opa1
WT LT-HSC
Abin1Q478H/Q478H LT-HSC
WT HPC-2
Abin1Q478H/Q478H HPC-2
WT MPP
WT HPC-1
Abin1Q478H/Q478H HPC-1
Abin1Q478H/Q478H MPP
